# DNaseI Hypersensitivity and Ultraconservation Reveal Novel, Interdependent Long-Range Enhancers at the Complex Pax6 Cis-Regulatory Region

**DOI:** 10.1371/journal.pone.0028616

**Published:** 2011-12-29

**Authors:** David J. McBride, Adam Buckle, Veronica van Heyningen, Dirk A. Kleinjan

**Affiliations:** Medical Research Council Human Genetics Unit, Medical Research Council Institute of Genetics & Molecular Medicine, University of Edinburgh, Western General Hospital, Edinburgh, United Kingdom; Institute of Cellular and Organismic Biology, Taiwan

## Abstract

The *PAX6* gene plays a crucial role in development of the eye, brain, olfactory system and endocrine pancreas. Consistent with its pleiotropic role the gene exhibits a complex developmental expression pattern which is subject to strict spatial, temporal and quantitative regulation. Control of expression depends on a large array of cis-elements residing in an extended genomic domain around the coding region of the gene. The minimal essential region required for proper regulation of this complex locus has been defined through analysis of human aniridia-associated breakpoints and YAC transgenic rescue studies of the mouse *smalleye* mutant. We have carried out a systematic DNase I hypersensitive site (HS) analysis across 200 kb of this critical region of mouse chromosome 2E3 to identify putative regulatory elements. Mapping the identified HSs onto a percent identity plot (PIP) shows many HSs correspond to recognisable genomic features such as evolutionarily conserved sequences, CpG islands and retrotransposon derived repeats. We then focussed on a region previously shown to contain essential long range cis-regulatory information, the Pax6 downstream regulatory region (DRR), allowing comparison of mouse HS data with previous human HS data for this region. Reporter transgenic mice for two of the HS sites, HS5 and HS6, show that they function as tissue specific regulatory elements. In addition we have characterised enhancer activity of an ultra-conserved cis-regulatory region located near Pax6, termed E60. All three cis-elements exhibit multiple spatio-temporal activities in the embryo that overlap between themselves and other elements in the locus. Using a deletion set of YAC reporter transgenic mice we demonstrate functional interdependence of the elements. Finally, we use the HS6 enhancer as a marker for the migration of precerebellar neuro-epithelium cells to the hindbrain precerebellar nuclei along the posterior and anterior extramural streams allowing visualisation of migratory defects in both pathways in Pax6*^Sey/Sey^* mice.

## Introduction

Mechanisms that control gene expression ensure that the information contained in the genome is correctly utilised. For genes with a complex expression pattern this control is exerted, at least in large part, by *cis-*acting DNA sequences such as promoters, enhancers, insulators and boundary elements (e.g. [1], [2]). The action and interplay of all these elements is required to drive expression of the gene in the appropriate spatiotemporal pattern. Thus it is important to fully define and characterise the regulatory elements that act on a gene to properly understand how the correct expression pattern is achieved, and how its disruption may lead to disease [3], [4].

Pax6, a paired and homeodomain containing transcription factor, is a developmental regulator with a strictly controlled expression pattern. Expression is found in all structures of the developing eye, in regions of the forebrain, hindbrain, cerebellum and spinal cord, the olfactory system and in pancreatic islet cells [5]–[10], and the gene is crucial to the correct development of these tissues [11]–[18]. Tight regulation of Pax6 transcription is important not just to generate a correct spatio-temporal expression pattern, but also to enforce the correct level of gene expression. Heterozygous disruptions in the *PAX6* gene cause the human eye malformation aniridia [19] and the mouse *smalleye* (*Sey*) mutation demonstrating dosage sensitivity [20], [21]. Brain defects have also been observed in PAX6 haplo-insufficient aniridia patients [22]. Overexpression of the gene also causes eye and brain malformations [23]–[26], indicating that Pax6 dosage is critical for correct development of these tissues.

Control of expression depends on a large array of cis-elements residing in an extended genomic domain around the coding region of the gene [27]–[33]. The role of distal *cis-*acting DNA elements in the control of *Pax6* expression was initially suggested by the discovery of a number of aniridia patients with two wild type *Pax6* coding alleles, but carrying breakpoints far downstream of the gene [34]–[36]. The role of this downstream region in *Pax6* expression was studied further using *smalleye* (*Sey*) mice. Introducing a 420 kb YAC containing the human *PAX6* locus into *Sey* mice rescues the phenotype [23]. However phenotypic rescue is only achieved when sequences far downstream of the *Pax6* coding sequence are included, as a shorter 310kb YAC lacking these sequences was not able to rescue, showing their presence is critical for *Pax6* expression [30].

A number of studies have characterised enhancers in the *Pax6* downstream region, with the majority focussing on evolutionarily conserved DNA sequences [30]–[33], [37]. Evolutionarily conserved regions are apparent when performing *in-silico* multi-species comparative genomic analysis of a genomic locus [38]. The disadvantage of a comparative genomics approach is that important cis-regulatory regions that are species specific or not recognisably conserved for other reasons will be overlooked [39]. Furthermore, a certain evolutionary distance is necessary between the compared species for conserved fragments to stand out from random sequence similarity, which will consequently overlook non-deeply conserved functional elements [38]. The approach also fails to provide information on potential tissue-specific activity of the putative regulatory element. We have used DNase hypersensitive site mapping to carry out an unbiased, locus wide search for novel cis-regulatory elements in the *Pax6* critical genomic region. DNase I hypersensitive sites (HSs), which are believed to present as a consequence of the remodelling or removal of nucleosomes by DNA binding proteins, leaving the surrounding DNA preferentially accessible for degradation by DNase I, are a marker for active cis-regulatory sequences [40], [41].

By mapping our results onto the output of a comparative genomics program (a percent identity plot (PIP) [42] we were able to map many of the identified HSs to identifiable genomic features, such as promoters and evolutionary conserved DNA sequences. Analysis of the data confirms that for the Pax6 locus in general, guidance by evolutionary sequence conservation can be used as a reliable strategy in identifying putative regulatory elements. However, our results show that HS mapping also identifies novel cis-regulatory elements that lack ‘deep’ conservation to evolutionarily more distant genomes, and which would therefore likely have escaped investigation for enhancer activity.

HS site analysis of a particular region of the locus, the DRR (Downstream Regulatory Region) has been carried out previously in a human lens derived cell line (CD5a) [30]. Thus we were able to compare HSs identified in different species. This comparison suggested that some HSs in this region were species specific, or alternatively could reflect differences between cell lines even if derived from equivalent tissues.

We tested the ability of a fragment containing a HS site found in the human CD5a but not in the mouse MV+ lens cell line, to drive reporter gene expression in transgenic mice. Strong expression was found in the optic cup, diencephalon and spinal cord. We next tested a strong tissue-specific HS found in mammals but lacking deeper sequence conservation, HS6, and show strong expression driven by this element in the rhombic lip and precerebellar nuclei in the hindbrain. Additionally we characterise reporter expression driven by an ultraconserved region located downstream of the Pax6 gene and show it to drive a complex expression pattern which overlaps with the HS5 pattern in the optic cup and with the HS6 driven pattern in the rhombic lip and precerebellar nuclei. Considering the overlap in expression sites between these cis-elements we used a collection of YAC reporter transgenic and Pax6 mutant lines to determine functional interrelationships between the elements and their dependence on expression of *Pax6* itself. This analysis suggests HS6 is essential for Pax6 expression in the precerebellar nuclei and acts independently of Pax6 expression, while HS5 activity depends on Pax6 but is nevertheless essential for expression in the optic cup and diencephalon P1 region. The E60 enhancer region is unable to drive Pax6 expression in the absence of the DRR (downstream regulatory region, including HS5 and HS6) in the optic cup, diencephalon and precerebellar neuroepithelium. These findings provide further insight into the complex cis-regulatory control mechanisms of this important developmental regulator.

## Results

### DNase I HS site analysis of the Pax6 locus

**Figure 1:**
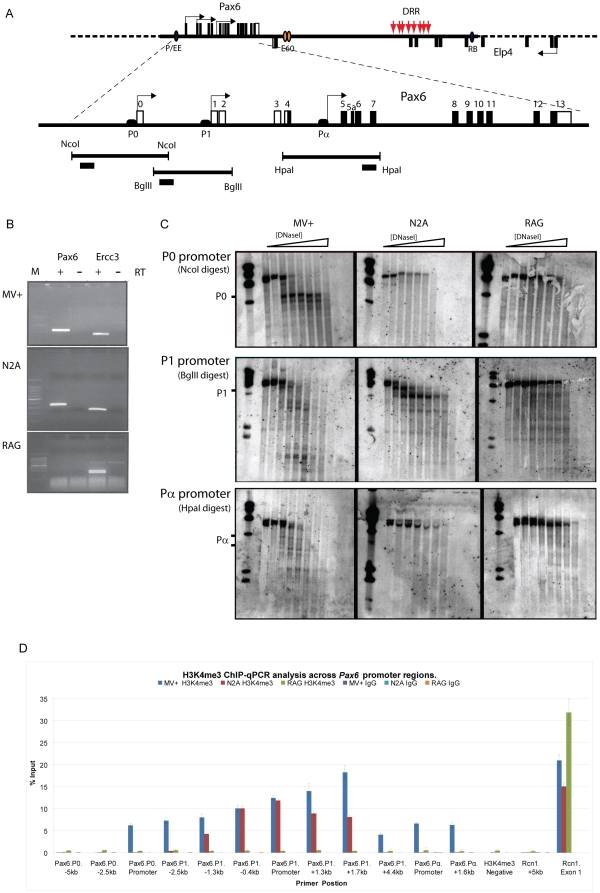
DNase I hypersensitive site mapping over the *Pax6* promoter regions. DNaseI HS mapping was carried out in three different mouse cell lines, MV+ (lens derived), N2A (neuroblastoma derived) and RAG (embryonic kidney derived). A) Schematic overview of the *Pax6* genomic locus on mouse chromosome 2E3. The extent of the locus examined by HS mapping is indicated by the solid portion on the dashed line. The exons of *Pax6* and the adjacent Elp4 gene are shown as black rectangles. Blue and orange ellipses indicate the positions of some known enhancers, and red arrows show the position of previously documented hypersensitive sites in the human *PAX6* downstream regulatory region (DRR). Below the proximal *Pax6* region is shown enlarged, with the exons indicated by numbered rectangles (open rectangle for non-coding exons, black for coding exons). Positions of the P0, P1 and Pα promoters are shown. The restriction fragments analysed for hypersensitivity and probe positions are indicated. (B) Expression analysis of Pax6 in the cell lines used in this study. rtPCR for Pax6 confirms expression in MV+ and N2A, and absence of Pax6 mRNA in RAG. The ubiquitously expressed Ercc3 was used as control. (C) HS mapping Southern blots of the *Pax6* promoter region. Hypersensitive sites are found in the promoter regions of *Pax6* in the expressing cell lines MV+ and N2A, but not in the Pax6 negative RAG cells. HS sites are found in the P0, P1 and Pα promoters in the MV+ cell line, while the N2A cell line only shows hypersensitivity in the P1 promoter. Positions of transcription start sites are marked by the side of the blot. (D) Chromatin immuno precipitation for tri-methylated histone H3K4, a mark of active chromatin, demonstrates the three known *Pax6* promoters, P0, P1 and Pα are all active in MV+. In N2A only P1 carries the active H3K4me3 mark, while none of the promoters carry the H3K4me3 mark in RAG cells. The ubiquitously expressed reticulocalbin (Rcn1) promoter was used as positive control and an intergenic sequence lacking any known features was used as negative control.

DNase I HS site analysis was performed across a region of mouse chromosome 2E3 ranging from 20 kb upstream of the Pax6 P0 promoter to approximately 170 kb downstream of the gene (from mm9/NCBI37 position chr2:105,495,760 to chr2:105,678,886) (Figure 1A). This region has previously been shown to be critical for Pax6 expression [36], [43], [30], [33]. To identify putative tissue-specific regulatory elements we scanned the region for HS sites in cell lines derived from lens (MV+ [44]) and brain (N2A [45]) tissue origins. Pax6 plays a major role in development in both these tissues and cell lines provide a uniform and readily available source of chromatin. Pax6 expression was confirmed by rtPCR (Figure 1B) and immunohistochemistry (not shown). For comparison a Pax6 negative cell line, the kidney derived RAG cell line [46] was included in the assay. DNase I HS analysis was carried out by a conventional Southern blotting based strategy [40], [47]. By analysing 96 hybridisations, we identified 44 HSs in the MV+ cell line, 33 in N2A and 28 in RAG (Table 1; Figures S1, S2, S3, S4, S5).

**Table 1:** DNase I HS sites across the *Pax6* locus.

PIP position	DNase I HSs	Associated genomic element	Presence in cell lines		
			MV^+^	N2A	RAG
0		Chr2:105,495,760 (mm9/NCBI37)			
11.6	NR	Previously characterised enhancer		+	
12.5	HS12.5	Highly conserved sequence		+	
16	EE	Previously characterised enhancer	++		
20	P0	*Pax6* P_0_ promoter	++		
21.4	HS21.4	CpG / GpC >0.75	++	+	
23.9	HS23.9	CpG / GpC >0.75	++	++	
24.9	HS24.9	None		++	
27	P1	*Pax6* P_1_ promoter	++	++	
27.2	HS27.2	CpG / GpC >0.75	++	++	
33.5	NRE	Previously characterised enhancer	++		+
35.1	HS35.1	CpG / GpC >0.75	++		++
38.2	CE1	Previously characterised enhancer	++		
39.8	CE2	Previously characterised enhancer	++		
41.5	CE3	Previously characterised enhancer	+	++	
42.1	CE4	Highly conserved sequence	++	++	+
47.5		Pax6 stopcodon Chr2:105,536,510-2			
48	UTR	*Pax6* final exon	+	+	+
49	HS49	Highly conserved sequence			+
59.5	HS59.5	LINE1 element	++	++	++
61.0	HS61.0	SINE	++	++	++
61.7	HS61.7	LTR	++		++
62.4	HS62.4	LINE1 element	+		+
66.8	HS66.8	Highly conserved sequence	+	+	+
70	E60	Ultra conserved sequence	*	*	*
85	HS85	LINE1 element	++	++	++
90	HS90	Mammalian conserved sequence	+	+	+
91.1	HS91.1	SINE	++	++	++
95.5	HS95.5	LTR	+	+	+
104.9	E100	Previously characterised enhancer	+	+	+
107.9	HS107.9	SINE	+	+	+
108.1	HS108.1	SINE	+	+	+
122.2	HS122.2	SINE	+	++	
123.4	HS123.4	None	+	++	
123.9	HS123.9	CpG / GpC >0.6	+	+	+
125.4	HS125.4	CpG / GpC >0.6	++	+	+
129.8	HS129.8	Highly conserved sequence	+		
130.5	SIMO	Previously characterised enhancer	++		
131		SIMO element Chr2:105,619,600-105,620,100			
134.5	HS1	Previously identified HS site	++		
138.2	HS2	Previously identified HS site	++		
139	HS3	Previously identified HS site	+		
147.1	HS6	Previously identified HS site	++		
149	HS7	Previously identified HS site	++		
151.5	HS8	Previously identified HS site	+	++	++
167.1	HS167.1	LINE1 element	+	+	+
170	HS170	Mammalian conserved sequence	+	+	+
171	HS171	LINE1 element		+	+
172.7	HS172.7	CpG / GpC >0.6	+	++	+
174.1	HS174.1	None	++	++	++
183.5	HS183.5	None	++	++	++
185	RB	Previously characterised enhancer	++	++	++
188.7	HS188.7	LINE1 element	++	++	++
191.5		Elp4 exon 4 chr2:105,687,384-105,682,517			

The presence of DNaseI hypersensitive sites in the three cell lines used (MV+, N2A, RAG) at indicated locations in the Pax6 locus is shown by their position on the PIP. Associated features are indicated next to the name of the HS site.

To assess the validity of our HS mapping strategy we first examined the HS pattern around the Pax6 P0, P1 and Pα promoter regions. Consistent with the absence of Pax6 expression in the RAG cell line no HS sites were observed in any of the three promoter regions in this cell line, whilst the Pax6 expressing cell lines, MV+ and N2A, show DNase I hypersensitivity in the P1 promoter region (Figure 1C). Interestingly, the HS site pattern over the wider P1 promoter region differs between the cell lines. However, while the N2A cells show no hypersensitivity at either the P0 or Pα promoters, MV+ cells exhibit hypersensitivity at all three, suggesting N2A cells use only the P1 promoter while MV+ cells use the P0, P1 and Pα promoters.

To confirm this pattern of promoter usage in the three cell lines we performed Chromatin Immuno Precipitation (ChIP) for the H3K4me3 histone modification, known as a mark of active promoter regions [48]. Enrichment for the H3K4me3 mark was found over all three P0, P1 and Pα promoters in the MV+ cells, confirming their active status in this cell line. In contrast, in N2A cells H3K4me3 enrichment was found only over the P1 promoter, while being absent from the P0 and Pα promoters, in accordance with the HS data. Finally, no enrichment was found on any of the promoters in the non-expressing RAG cells (Figure 1D).

**Figure 2:**
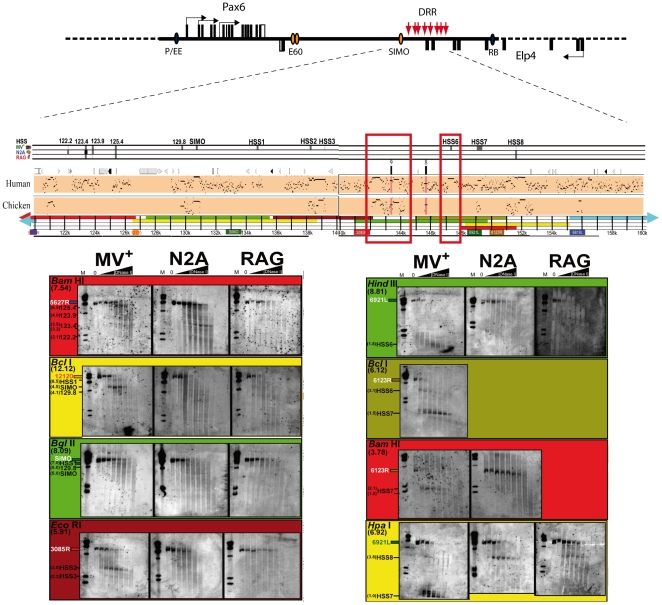
DNAse hypersensitivity blots covering the *Pax6* downstream regulatory region (DRR). A schematic diagram of the *Pax6* locus is shown with the DRR indicated. Sequence conservation patterns in the region are shown by Percentage Identity Plot (PIP) using pairwise comparison of mouse sequence against human and chicken. The restriction fragments analysed are indicated by a colour coded line underneath the PIP plot. The fragments used for probing the blots are also indicated. The DRR harbours several tissue-specific HS sites found in the MV+ cell line. These include two sites marking the previously characterized SIMO lens enhancer, a site marking the HS2/3 retinal enhancer and strong sites coinciding with the positions of HS sites previously found in the human *PAX6* locus, HS6 and HS7. An HS site at position 123.4 appears specific for N2A cells. A constitutive HS site, HS8, is found towards the distal end of the DRR in all cell lines examined, but appears strongest in N2A and RAG. Open red rectangles over the PIP plot show the extent and position of the HS5+ and HS6 containing fragments characterized in reporter transgenic mice.

Having confirmed the ability of HS mapping to successfully identify *Pax6* promoters, we next assessed whether the newly identified HS sites mapped to other recognisable features in the locus. As a first step we mapped the HS sites onto a Percentage Identity Plot (PIP) of the *Pax6* locus [42], using the mouse locus as the baseline sequence with pairwise comparisons to the human and chicken *Pax6* loci (Figure 2; Figures S1, S2, S3, S4, S5). PIPs provide a graphical representation of sequence conservation between two species. By drawing comparisons from different species in parallel, DNA sequences that are conserved across multiple species can easily be identified. Thus mapping HSs onto PIPs allowed us to determine whether HSs corresponded to multi-species highly conserved sequences. DNase I HSs are marked in the tracks above the PIP by a black or grey line, the intensity of which corresponds to the strength/frequency of the HSs in the cell population under analysis. HSs were found to map to 50 distinct positions across the locus. Many of these have previously been characterised (EE, P0, P1, NRE, 7CE1-4, E60A, E100, SIMO, HS2, 3, RB) as *Pax6* regulatory elements in transgenic studies [27]–[33], [37]. DNase I HS mapping has previously been carried out for a 30 kb fragment located >150kb downstream of the *PAX6* coding region (termed the downstream regulatory region; DRR), in a human lens epithelium derived cell line, CD5a [30]. In our current study we observe the presence of HS sites in murine MV+ cells at most positions previously identified in the human CD5a cell line, including HS1, 2, 3 and HS 6, 7, 8 [30]. However, two of the weaker HS sites (HS4 and 5) found in CD5a cells were not found here. In contrast, no HS site was observed at the SIMO element in human CD5a, but a strong site was found in the mouse MV+ cells. A fragment containing the SIMO element has been shown to be capable of driving reporter gene expression in the lens, diencephalon and hindbrain of developing mouse embryos [30], consistent with the observed hypersensitivity in the MV+ lens cells. It is currently unclear whether the difference in hypersensitivity at these sites represents a species-specific difference between the mouse and human elements, or whether it indicates that the lens epithelium derived CD5a and MV+ cell lines are not completely equivalent. Furthermore, we also identified HSs at positions 12.5, 21.4, 49.0, 66.8 and 129.8, and a seemingly general increase in DNaseI sensitivity over a wider region at the E60UCS ultra-conserved element. These sequences have not previously been characterised and are new potential *Pax6* regulators (Figure S1; Table S1).

### Transgenic analysis of the hypersensitive site HS5+ region

**Figure 3:**
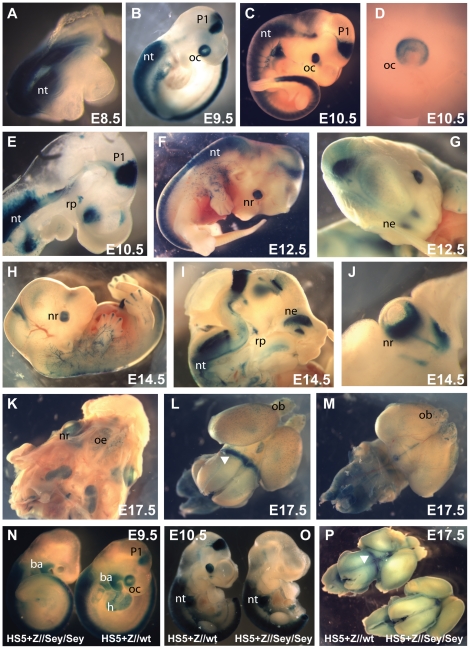
Characterisation of the HS5+ region in transgenic mice. Transgenic lines were generated with the micro-injection fragment depicted in Figure 2, and their LacZ expression pattern characterized throughout development. (A) At E8.5 staining is found along the neural tube (nt) up to hindbrain level, and this expression in the neural tube is maintained throughout development. (B–D, F) In addition to the neural tube, strong reporter expression is found in the prosomere P1 region of the diencephalon (P1) and in the developing optic cup (oc) from E9.5 onwards. (E) Embryo cut sagitally along the midline showing expression in the neural tube, diencephalon, optic cup, and also seen in Rathke's pouch (rp) in some of the lines. (G–I) LacZ staining is found in the olfactory epithelium (oe). (J) Strong expression is maintained in the neuroretina (nr) at E14.5. (K) Skull of an E17.5 embryo with the brain removed, showing expression in the eye and olfactory epithelium. (L) Dorsal view of an E17.5 brain showing expression in a lateral band around the thalamic area of the brain (white arrowhead). (M) Ventral view; patchy staining is seen in the olfactory bulbs (ob). (N–P) HS5+ function in eye and diencephalon is Pax6 dependent. At E9.5 no staining is seen in the eye or diencephalon of a HS5+Z//*Pax6* ^sey/sey^ transgenic embryo, while expression in the neural tube is unaffected. A HS5+Z//*Pax6* ^wt/wt^ littermate is shown alongside for comparison. Expression in the heart (h) and branchial arches (ba) in (N) is due to site of integration and specific to the transgenic line used, and is also not affected by *Pax6*/smalleye status. (O, P) A second, independent transgenic line tested at E10.5 and E17.5 confirms the Pax6 dependence of the HS5+ enhancer fragment in diencephalon and eye, but not in the neural tube.

Following the observed difference in hypersensitivity between the human and mouse lens cell lines, and considering the strong and deep sequence conservation of the region, we were curious to see whether a fragment containing the region including HS5 plus an adjacent highly conserved element, collectively called HS5+, would be capable of driving reporter gene expression in the mouse. We obtained several independent transgenic lines carrying this element linked to a hsp68-lacZ reporter cassette (6 expressing/9 total transgenic lines). Reporter analysis revealed consistent expression along the length of the spinal cord from E8.5 (Figure 3A) and in the developing optic cup and prosomere P1 region of the diencephalon from E9.5 onwards (Figure 3B, C, D). Strong LacZ staining was seen in the spinal cord from the level of rhombomere 6 towards the caudal end. In the diencephalon expression remains strictly limited to prosomere P1. From E12.5 staining is seen in the olfactory epithelium (Figure 3G, I, L), and is maintained in the eye and diencephalon (Figure 3F, H, J). Expression was also observed in Rathke's pouch [16], which develops into the anterior pituitary, in some of the lines (Figure 3E, I). At late gestational stages (E17.5) expression was found in a distinct band around the lateral sides of the thalamic area of the brain, and in the eye, olfactory epithelium and olfactory bulbs with some variation in strength of expression between the different lines (Figure 3K, L, M).

Pax6 expression in the diencephalon is known to be subject to auto-regulatory control [7]. A number of other cis-elements with enhancer activity in prosomere P1 have previously been identified in the *Pax6* locus and some have been shown to depend on Pax6 itself as their activity was lost in homozygous smalleye mice [32], [33]. To test whether the HS5+ enhancer region is also subject to Pax6 autoregulation we crossed a representative HS5+-Z reporter line onto a smalleye background and examined reporter expression in transgenic embryos homozygous for the smalleye mutation at E9.5, E10.5 and E17.5. LacZ expression in the spinal cord appeared unaffected by the absence of Pax6, but expression in the eye and P1 prosomere was completely abrogated indicating strong autoregulation of the eye and diencephalon components contained within the HS5+ fragment (Figure 3N, O, P).

### Characterisation of the mammalian specific HS6

**Figure 4:**
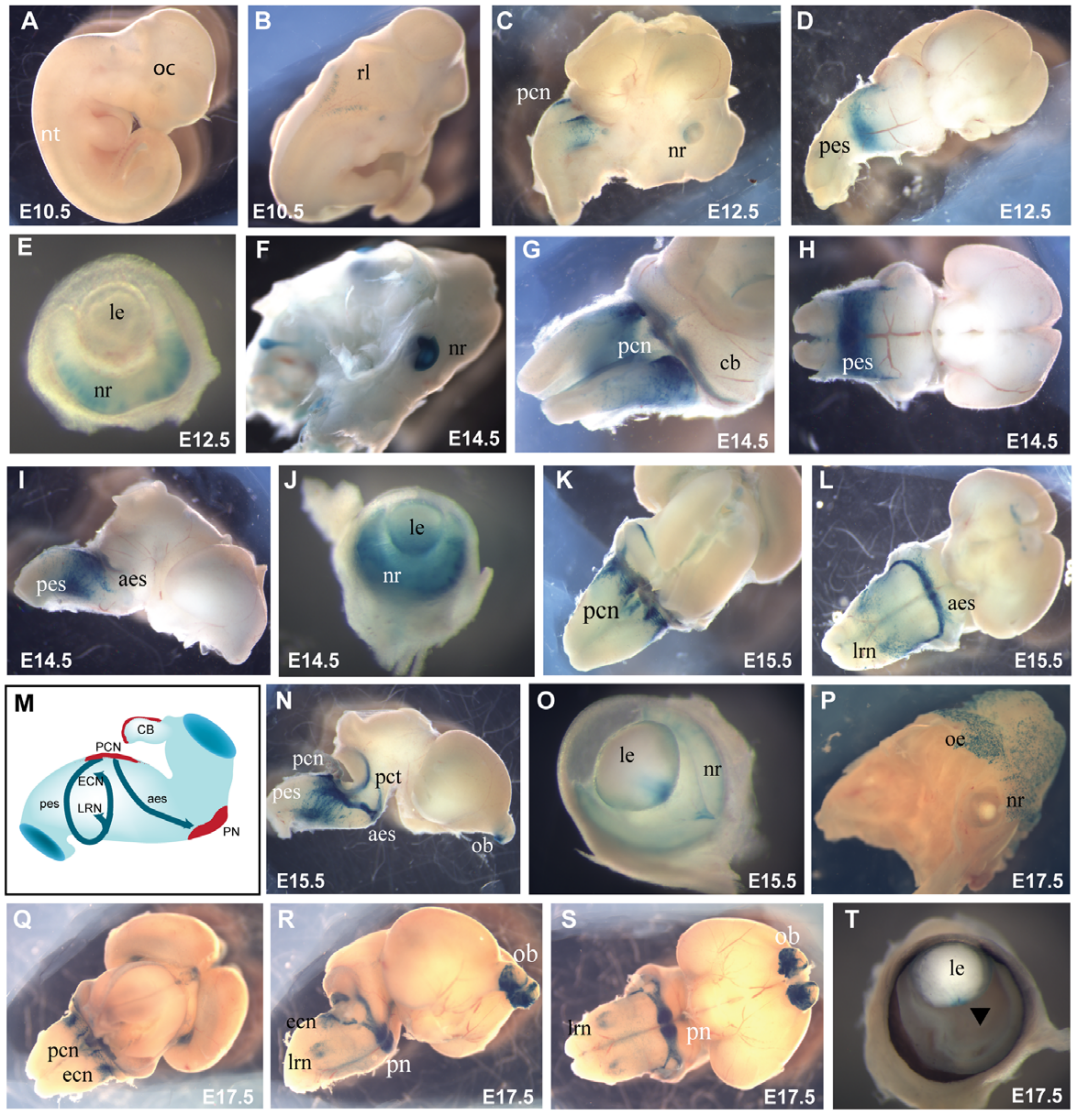
Expression pattern driven by the HS6 element. (A, B) No distinct expression pattern that was consistent between the lines was found at E9.5 and E10.5. (B, C) The earliest specific pattern appeared at the rhombic lip (rl) at E10.5 in the strongest expressing lines, and was apparent in other lines from E12.5. (D) At E12.5 expressing cells in the precerebellar nuclei (pcn) at the rhombic lip start to migrate along the posterior extramural stream (pes). (F) At E12.5 expression also appeared in the eye and olfactory epithelium (oe). (G, K, Q)Expression in the pcn continues throughout development, and cells migrate from the pcn to settle in the precerebellar nuclei of the pons. (D, H, L, N) Staining is seen in the posterior extramural stream (pes) from E12.5 until E15.5, and in the anterior extramural stream (aes) starting from E14.5 continuing to E17.5 (I, L, R, S). (J) Expression in the eye is strongest at E14.5 and found in the neuroretina (nr), the hyaloid vasculature and the proximal tip of the lens (le). (M) Schematic diagram showing the tangential migration streams followed by migrating neurons from the precerebellar neuron-epithelium (pcn) towards their targets in the lateral reticulate nuclei (lrn), external cuneate nuclei (ecn) and pontine gray nuclei (pn). (O) From E15.5 expression in the eye declines, and (T) by E17.5 staining is only seen in the proximal tip of the lens. Expression in the olfactory epithelium and olfactory bulbs is maintained throughout development. (N) At E15.5 both the late stage posterior extramural stream (pes) and the early stage anterior extramural stream (aes) are staining, and the ponto-cerebellar tract (pct) also appears. (Q, R, S) Reporter xpression continues in the pcn, and in the external cuneate nuclei (ecn) and lateral reticulate nuclei (lrn). At E17.5 cells migrating along the aes have settled in the pontine gray nuclei (pn) and are strongly positive.

In contrast to the HS5 region which is highly conserved across many distantly related vertebrate species (deep conservation), but does not show strong hypersensitivity in the cell lines tested, the adjacent HS6 region produces a strong tissue-specific hypersensitive site in the MV+ cells (Figure 2), but lacks deep sequence conservation, such that the element is not detectable by conservation in comparisons with chicken (Figure 2), frog or any other non-mammalian species tested. Clear conservation is detected in genomic sequence from all mammalian species analysed, including platypus, wallaby, possum and dolphin (Figure S6). Intrigued by the strong hypersensitivity and lack of deep sequence conservation we proceeded to test the HS6 region in our transgenic reporter assay. The LacZ reporter driven by the HS6 fragment was not active at early stages of development (Figure 4A), but drives a very distinct expression pattern at later gestational stages. Activity starts from around E10.5 at the dorsal edges of the rhombencephalon (Figure 4B). Expression in this region increases over the next period during formation of the rhombic lip (rl). At E12.5 staining is observed in the pre-cerebellar neuroepithelium (pcn), and in a stream of cells that migrate tangentially along the outside of the pontine region, constituting the early stages of the posterior extramural stream (pes) [17], [49]. (Figure 4C, D). A subset of neurons from the pcn migrates along the pes from the rhombic lip crossing the midline towards the contra-lateral side of the medulla to settle in the lateral reticular nuclei (lrn) and external cuneate nuclei (ecn) [17], [49], [50]. Expression also starts in the nasal epithelium and neuroretina (Figure 4 E, F). By E14.5 expression is strong in the precerebellar neuroepithelium (pcn) and the posterior extramural stream (pes) has become a wide stream (Figure 4G, H). Some blue cells are starting to migrate along the anterior extramural stream (aes) (Figure 4I). At E15.5 the anterior extramural stream (aes) becomes very distinct and reaches the position for the pontine gray nuclei. The pcn and aes remain strongly positive at E17.5, but the pes is no longer visible at this stage (Figure 4Q, R). Expression in the pontine gray nuclei themselves and in the ponto-cerebellar stream is clear (However, the LacZ staining pattern suggests that the expression driven by the HS6 enhancer region does not extend to the reticulogeniculate nuclei just beyond the pontine gray nuclei (not shown). In addition to the distinct expression pattern in the precerebellar region of the transgenic embryos, HS6 shows activity in the olfactory bulbs and olfactory epithelium in several lines, albeit at varying levels of intensity between the transgenic lines (Figure 4F, N, P). HS6 also drives expression transiently in the developing eye during a limited period from E12.5 until E16.5. At E10.5 no expression is found in the eye, but by E12.5 clear staining is seen in the optic cup/neuroretina which peaks around E14.5. At this stage some X-gal staining is also seen in the hyaloid vasculature, the lens capsule and the posterior end of the lens at the site of attachment of the hyaloids artery (Figure 4J, O), but this staining is thought to be due to endogenous activity. Staining in the retinal layers has disappeared by E17.5, but some staining is still seen in the posterior end of the lens (Figure 4T).

### E60 is a large ultraconserved element

**Figure 5:**
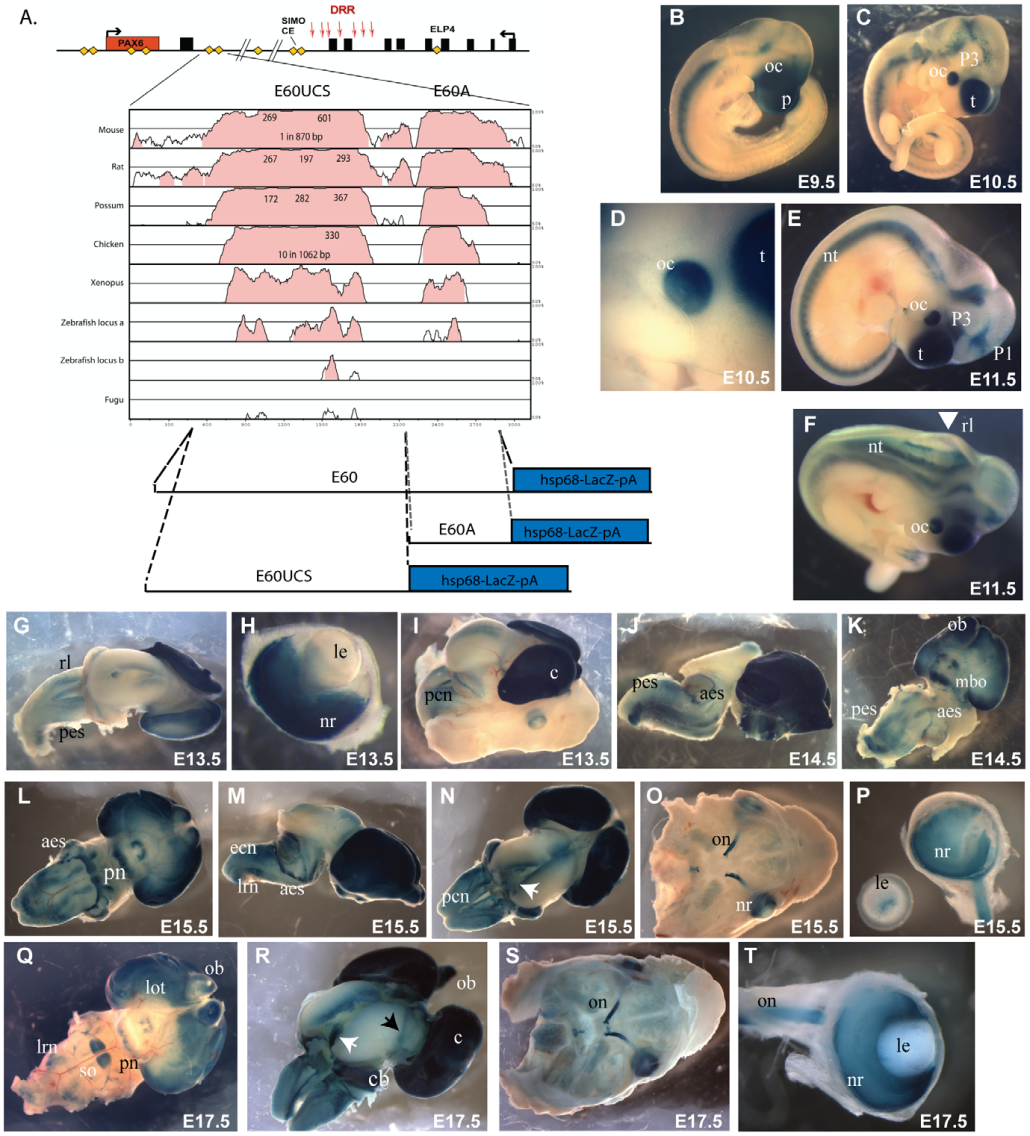
The E60 element is an ultraconserved enhancer driving a complex expression pattern. A) The E60 ultraconserved sequence (E60UCS) is located 25kb downstream of the *PAX6* stop codon in the human locus, in the large final intron of the adjacent ELP4 gene. The *PAX6* coding region is shown as a single red rectangle; The ELP4 exons, transcribed from the opposite strand, are shown as black rectangles;. The positions of hypersensitive sites in the human DRR (downstream regulatory region) are indicated by red arrows. Yellow diamonds show previously characterized enhancers. An annotated VISTA plot demonstrates the extra-ordinarily high level of sequence conservation over a wide range of species. Numbers within the peaks show the length of stretches of 100% sequence identity with the human element. The E60UCS contains several stretches of perfect sequence conservation between human and mouse, possum and chicken and is also highly conserved to xenopus. (B-T) Reporter transgenic characterization of the full E60 element. (B) At E9.5 expression is found in the optic cup (oc) and prosencephalon (p). (C, D) At E10.5 staining is seen in the telencephalon (t), optic cup (oc) and prosomere P3 of the diencephalon. (E, F) In addition some staining is found in the prosomere P1 region at E11.5. No staining is seen at the rhombic lip (rl) at this stage. (F) (G, H, I) At E13.5 staining is observed in the precerebellar neuroepithelium at the rhombic lip (lrl), and weakly in the posterior extramural stream (pes). Strong staining is seen in the telencephalon and in the neuroretina (nr), but not the lens (le). (J, K) At E14.5 expression has become stronger in the precerebellar neuroepithelium (pcn) and the posterior extramural stream (pes). Migration along the anterior extramural stream (aes) has started. The olfactory bulbs (ob) have started to form and are also positive. Staining is also seen at the position of the mammillary body (mbo). (L, M) Expression continues along the aes, and positive cells are settling in the pontine gray nuclei (pn). Strong signal is found in the cortical lobes (co), the olfactory bulbs (cb). (N) Additionally staining is present in the midbrain tegmentum at the position of the dorsal tegmental nuclei (white arrowheads). (O) A E15.5 skull with the brain removed shows expression in the eyes, specifically (P) in the neuroretina (nr) and optic nerve (on). A patch of staining at the proximal side of the lens (le) is thought to be a staining artefact. (Q) At E17.5 staining is seen in the pontine gray nuclei (pn), lateral reticular nuclei (lrn), cortical lobes, olfactory bulbs and in the lateral olfactory tract (lot). (R) A dorsal view of a dissected E17.5 brain cut open along the roof midline of the mid- and hindbrain shows expression in the dorsal tegmental nuclei (white arrowhead) as well as in the external granular layer of the cerebellum (cb). (S, T) Expression in the neuroretina and optic nerve is shown in an E17.5 skull and dissected eye.

Multi-species sequence conservation analysis highlights the presence of a large number of conserved elements in the *Pax6* locus (e.g. [33]). Among these the E60 ultraconserved element (E60UCS, previously named E60B) is a putative cis-regulatory sequence with extreme sequence conservation across many species. The E60UCS is located 25kb downstream from the *Pax6* 3′UTR in the large final intron of the adjacent ELP4 gene, and exhibits 94% nucleotide sequence identity over a 1400 bp region between human and mouse. Applying the definition for ‘ultraconservation’ of absolute sequence conservation between human, mouse and rat over a length of >200 bp [51] the E60 element qualifies as one of the most extreme ultraconserved elements (UCSs) in the genome, containing a stretch of 601 basepairs of complete identity between human and mouse (Figure 5A). Comparison with the chicken *Pax6* locus also reveals extreme conservation over >1kb region, including a 301 bp length stretch of 100% identity, and the element is also highly conserved to possum, frog, and the zebrafish *Pax6*a locus (Figure 5A). While no typical, well-defined DNase HS was observed at the E60 region in any of the three cell lines, the HS blots suggested a wider stretch of increased general DNase sensitivity at the E60 element in the Pax6 expressing MV+ and N2A cell lines (Figure 2). Located immediately adjacent to the E60UCS lies another highly and deeply conserved sequence, which we named E60A. To investigate the function of this large highly conserved region we generated transgenic mice with a 2.3 kb fragment, E60Z, containing both the E60UCS and adjacent E60A conserved elements (3 expressing/4 total transgenic lines). In addition we made a number of transgenic mice with only the E60UCS fragment (3 expressing/5 total transgenic lines), and also produced transient E16.5 transgenic embryos with the E60A region only (4 expressing/ total not determined), as described previously for earlier stage embryos [36].

The expression pattern driven by the E60Z fragment is shown in Figure 5. In the early embryonic stages (E9.5 – E11.5) the expression pattern of the full E60Z construct mirrors the pattern of the previously described E60A subfragment only transients [36], with expression in the optic cup and stalk, the telencephalon and prosomere P3 of the diencephalon (Figure 5B–E). A low level of staining is also seen in prosomere P1. Notably, at E11.5 no expression is yet detected in the rhombic lip (Figure 5F, arrowhead). Expression in the precerebellar neuroepithelial cells at the rhombic lip appears from around E12.5 onwards. Strong staining is maintained in the telencephalon (Figure 5G–N), and also in the neuroretina of the eye (Figure 5H, I). The staining in the P1 prosomere is now seen around the lateral sides of the diencephalon, similar to staining seen with the HS5 element, but less strongly. At E14.5 the cells migrating along the pes are staining, as well as the first appearance of cells following the aes (Figure 5 J, K). Strong expression can be seen in the telencephalon and the olfactory bulbs, as well as in a region thought to be the mammillary body (mbo), not a known site of Pax6 expression (Figure 5K, L). This pattern of reporter expression is continued at late embryonic stages (E15.5–E17.5; Figure 5L–T)). At E15.5 staining of the aes becomes very conspicuous (Figure 5L, M). Staining of the pes is waning, but expression is now seen in the lateral reticulate nuclei (lrn) and external cuneate nuclei (ecn). Expression continues in the pcn, and staining can now also be seen in the cerebellum. Expression also continues in the eye and optic nerve (Figure 5O, R, S, T). At E17.5 there is clear staining of the olfactory bulbs, the lateral olfactory tract (lot), the cortical lobes, the external granular layer of the cerebellum, and the pontine grey nuclei (Figure 5Q, R). In the eye expression is seen in the neuroretina and optic nerve (Figure 5S,T).

**Figure 6:**
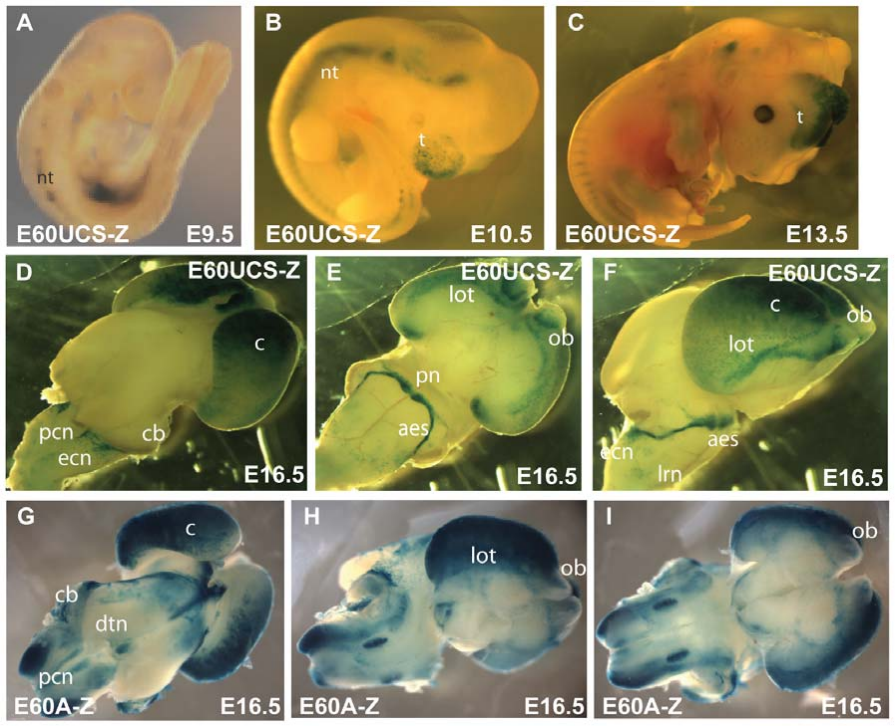
Expression patterns of the E60UCS and E60A elements. (A–F) Expression driven by the E60 ultraconserved element (E60UCS). (A) At E9.5 no specific expression is detected. Staining at the gonadal area is due to integration site of the construct and not seen in other lines. (B) Earliest specific expression starts from E10.5 in the telencephalon and has become very prominent by E13.5 (C). (D, E, F) At E16.5 expression is found in the cortical lobes (c), the olfactory bulbs (ob), the lateral olfactory tracts (lot) and the anterior extramural stream (aes) migrating from the precerebellar nuclei (pcn) to the pontine gray nuclei (pn). Weaker staining can also be seen in the lateral reticulate (lrn), external cuneate nuclei (ecn), and the ponto-cerebellar tract (pct). (G–I) Expression pattern driven by the E60A element. (G) The E60A element drives strong expression in the cortical lobes (c), dorsal tegmental nucleus (dtn), and more weakly in the cerebellum (cb) in E16.5 transient transgenic embryos, but no specific expression is observed in the precerebellar neuro-epithelium (pcn). (H) A lateral view shows strong staining in the cortical lobes, the olfactory bulbs (ob) and the lateral olfactory tracts (lot). (I) Staining is clearly absent from the precerebellar migratory streams in a ventral view of a dissected brain.

As the early pattern of the full E60Z construct was the same as found previously for E60A transient embryos [36] we decided to look at the pattern driven by the E60UCS sequence on its own (Figure 6). This confirmed that the early expression is contributed by the E60A region. No consistent expression was found in E60UCS transgenic embryos at E9.5 apart from some staining in the neural tube due to the hsp68 promoter (Figure 6A, B). Expression in the gonadal region in Figure 6A and limbs in Figure 6B is due to site of integration of the reporter and not found in other lines with this construct. The E60UCS drives enhancer activity in the telencephalon starting weakly from E10.5 and increasing in strength towards E13.5 (Figure 6C). Telencephalic expression is then maintained throughout development (Figure 6D, E, F). At E16.5 expression is seen weakly in the final stages of migration of the pes, in the lrn and ecn, and strongly in the aes migrating to the pontine grey nuclei (pn). Reporter expression has continued in the telencephalon with strong staining of the cortical lobes, and in the lateral olfactory tracts (lot) projecting from the olfactory bulbs, which also show regions of strong expression (Figure 6E, F).

To determine whether the E60A element is only active at the early embryonic stages as described previously (Kleinjan et al., 2008), or also contributes to the later expression pattern we analysed transient transgenic embryos at E16.5. LacZ staining is seen in the cortical lobes, olfactory bulbs, lateral olfactory tracts (lot), and cerebellum, but not in the pcn, migratory streams or precerebellar nuclei (Figure 6G, H, I).

### HS6-Z as a marker for pcn migration in Pax6 deficient and overexpressing mice

**Figure 7:**
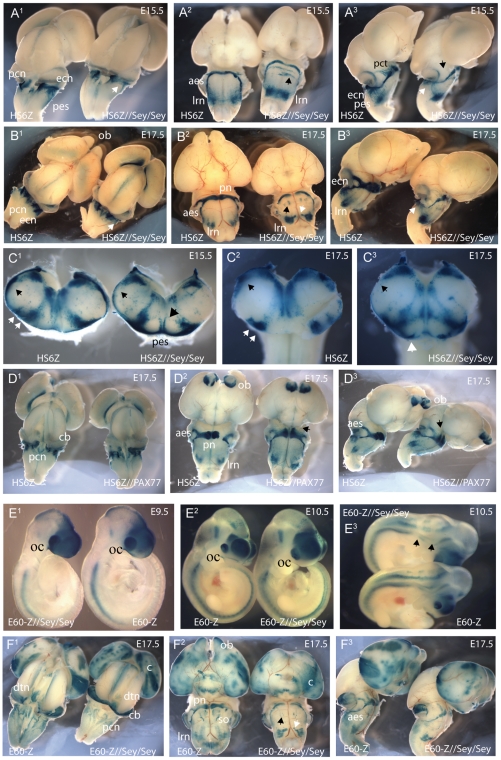
Enhancer activity of the HS6 and E60 elements in the absence of functional Pax6. Enhancer driven reporter activity was compared between *Pax6* ^sey/sey^ homozygous embryos and wildtype littermates in E15.5 and E17.5 dissected brains in dorsal (A^1^–F^1^), ventral (A^2^–F^2^) and lateral view (A^3^–F^3^). (A^1–3^) The HS6 element is functional in *Pax6* ^sey/sey^ mutant embryos. HS6 driven expression is seen in the precerebellar neuroepithelium (pcn), the anterior extramural stream (aes), and the external cuneate (ecn), lateral reticulate (lrn) and pontine gray (pn) nuclei. In *Pax6* ^sey/sey^ embryos staining is absent at the position of the ecn (white arrow), whereas staining is increased and disorganized in the lrn. A ventral view of transgenic embryos on wildtype and *Pax6* ^sey/sey^ background at E15.5 shows delayed migration of the pes across the midline. In wildtype embryos the majority of cells have crossed the midline and moved along to the lrn or ecn, while in *Pax6* ^sey/sey^ many LacZ positive cells are still near the midline. Migration along the aes is reduced and a subset of cells can be seen to follow aberrant migratory pathways (black arrow). No staining is seen in the ponto-cerebellar tract (pct, black arrow). (B^1–3^) At E17.5 the absence of staining in the ecn is clear (white arrow), as well as the reduced signal in the pontine nuclei (pn). In wildtype embryos the pct is strongly positive, but little signal is seen in the pct of *Pax6* ^sey/sey^ embryos (black arrow). LacZ staining also reveals cells mislocalised near the superior olive as a result of aberrant migration (black arrow). Staining is increased in the lrn which appears disorganized and shows a tail of positive cells towards the inferior olive. (C^1–3^) Coronal sections through E15.5 and E17.5 hindbrains. HS6Z marker activity is observed in the pcn, ecn and the characteristic double stripe pattern of the lrn in a wildtype littermate. In a *Pax6* ^sey/sey^ embryo staining is absent from the ecn (white arrow), but increased at the disorganized lrn which no longer has a double stripe appearance. Delayed migration of the pes results in strong signal at the ventral midline (arrow), with some cells appearing to migrate upwards along the midline (arrow). (D^1–3^) Expression of the HS6Z transgene in Pax6 overexpressing embryos at E17.5 shows a migratory defect of the aes, with the aes spreading out wider upon reaching the pontine gray nuclei and cells settling rostally from the pn (E–F) Expression driven by the full E60 region is maintained in *Pax6* ^sey/sey^ embryos. (E^1–3^) Comparison of wildtype and *Pax6* ^sey/sey^ embryos shows expression is maintained in the absence of functional Pax6 in all E60 positive tissues at E9.5 and E10.5, except for absence of staining in rhombomeres 3 and 5 of the hindbrain (black arrows). (F^1–3^) At E17.5 LacZ staining is found in the cortex (c), cerebellum (cb), dorsal tegmental nucleus (dtn) and precerebellar neuroepithelium (pcn) in both wildtype and *Pax6* ^sey/sey^ embryos. The reason for the patchy staining in the cortex is unclear. Consistent with the migratory defects in *Pax6* ^sey/sey^ embryos described using the HS6Z marker, staining is reduced in the anterior extramural stream (aes) and pontine gray nuclei (pn) and not seen in the pct (black arrow). The disorganized structure of the lrn is also marked by the E60Z reporter, including the ectopic tail from the lrn to the inferior olive (white arrowhead). The mislocalised LacZ positive cells near the superior olive (black arrowhead) are just visible with the E60Z marker. Weak expression is found in the superior olive (so) in wildtype embryos, but not in *Pax6* ^sey/sey^. (F^1–3^)

Previous studies of *smalleye* mice have shown that transcription of *Pax6* in the rhombic lip and precerebellar neuroepithelium is maintained in the absence of functional Pax6 protein [17]. However, lack of functional Pax6 is known to cause migratory defects of the pes in *Pax6* ^sey/sey^ embryos [50], [52]. As shown in Figure 4 the HS6-Z transgene is highly specific for the pcn and migratory streams (aes, pes), and hence could make a useful marker for these structures in mutant transgenic or knock-out mice. As proof of principle we studied LacZ staining in representative HS6 reporter lines when crossed onto *Pax6* mutant backgrounds. We first tested the effect of Pax6 deficiency. Inspection of E15.5 and E17.5 litters from HS6-Z//*Pax6* ^sey/+^ times *Pax6* ^sey/+^ crosses show that the HS6 enhancer is independent of Pax6 activity and remains active in homozygous *Pax6* ^sey/sey^ embryos (Figure 7A ^1–3^, B^1–3^). Strong reporter expression remains present in the pcn (Figure 7A ^1^, B^1^). At E15.5 clear differences are seen between wildtype and *Pax6* ^sey/sey^ embryos, consistent with the migratory defects of the pes seen in *ex-vivo* cultures of smalleye homozygous hindbrains [50]. Whereas in wildtype embryos positive cells of the pes have crossed the midline and are settling in the contra-lateral lateral reticulate nuclei (lrn) and external cuneate nuclei (ecn) (Figure 7 A^1–3^), in *Pax6* ^sey/sey^ embryos many cells of the pes are delayed near the midline. Staining in the lateral reticulate nuclei (lrn) at E15.5 and E17.5 indicates the disorganised structure of these nuclei in *smalleye* homozygotes. An increased staining level is seen at E17.5 in the lrn with a tail stretching to the position of the inferior olive (Figure 7 B^2^). No staining is seen at the position of the ecn of *Pax6* ^sey/sey^ embryos (Figure 7 B^1, 3^). Sections through the hindbrain at E15.5 show the migration delay, the absence of staining in the ecn and an increased number of LacZ positive cells appearing to migrate dorsally at the midline (Figure 7 C^2^). The HS6 enhancer is also a strong marker for the anterior extramural stream (aes). In accordance with a decrease in migratory capacity of these neurons in *Pax6* ^sey/sey^ embryos [17] staining intensity is greatly reduced in the anterior extramural stream (aes) of homozygous smalleye embryos (Figure 7B ^2^). In addition at E15.5 we also observe a bilateral pair of migration streams following an incorrect pathway. The staining pattern suggeststhat the cells cross the midline and settle in a mislocalised position near the superior olive, as seen at E17.5 (Figure 7 A^2^, B^2^). Staining of the ponto-cerebellar tract is also severely reduced in *Pax6* ^sey/sey^ embryos (Figure 7 B^3^). Next we examined the effect of Pax6 overexpression using the PAX77 YAC line carrying 5–6 additional copies of the (human) *PAX6* locus [23] to generate HS6Z/PAX77 double transgenic embryos. No differences were seen between HS6Z and HS6Z/PAX77 double transgenics at E15.5 (not shown). At E17.5 cells migrating along the anterior extramural stream (aes) have settled in the compact pontine gray nuclei in wildtype embryos, but in HS6Z/PAX77 double transgenic embryos the cells have spread wider extending towards the rostral side of the pons, indicating that overexpression of Pax6 affects migration of pcn cells along the aes (Figure 7D ^1–3^ black arrows).

### Autoregulation of the E60 enhancer region

We next investigated the dependence of expression driven by the E60 region on the presence of functional Pax6 by crossing a representative E60Z reporter transgenic line onto a homozygous smalleye background. Allowing for the morphological changes and migratory defects resulting from the loss of Pax6, the staining pattern in *Pax6* ^sey/sey^ embryos carrying the full E60Z transgene was similar to the pattern in *Pax6* ^wt/wt^ transgenic embryos. Expression in the eye, telencephalon and diencephalon was unaffected in smalleye embryos at E9.5 and E10.5 (Figure 7E ^1–2^).The only difference was in the hindbrain where reporter activity was no longer seen in rhombomeres 3 and 5 of *Pax6* ^sey/sey^ embryos (Figure 7E ^3^). At E17.5 staining is similarly maintained in most expression sites, though it is no longer visible in the ponto-cerebellar tract and it is also absent in the superior olive (Figure 7F ^1–3^). The reason for the patchiness of staining in the cortex is unclear.

### Interdependence of enhancer elements

**Figure 8:**
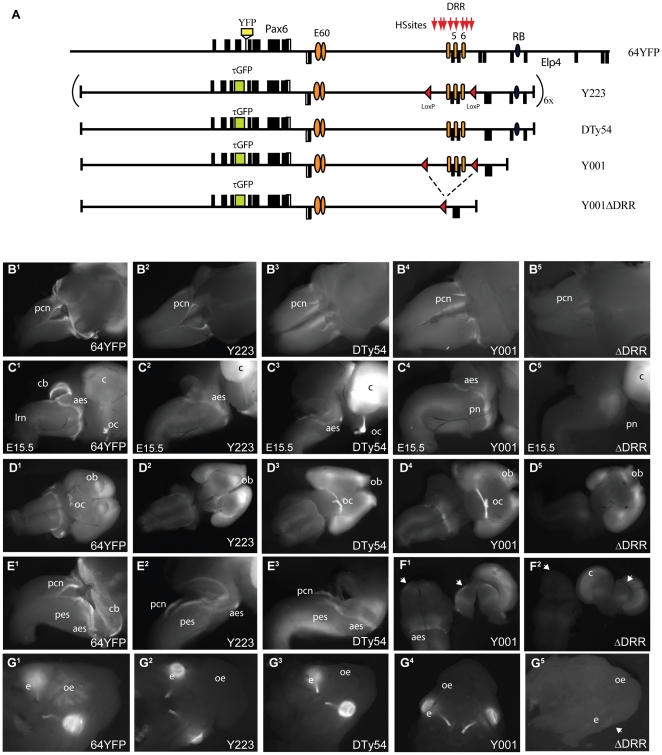
Examination of GFP fluorescence patterns in YAC transgenic mice at E15.5, in comparison with 64YFP, a YFP expressing targeted gene-trap allele for *Pax6*. (A) Schematic diagram of the *Pax6* genomic locus. *Pax6* exons are shown as black rectangles, exons of the adjacent Elp4 gene, transcribed from the opposite strand, are shown below the line. Location of the 64YFP knock-in cassette is shown, as well as the E60 elements (orange ellipses), the HS sites of the DRR and the HS5+ and HS6 elements (orange ellipses). The composition of the YAC reporter lines is shown below, including the multicopy full length Y223, single-copy full length DTy54, the single-copy line Y001 lacking the distal-most 20kb including the RB enhancer, and the DRR (downstream regulatory region) deletion line ΔDRR, which is directly derived from Y001 by Cre-mediated deletion of the 25 kb DRR region. The positions of the LoxP sites are indicated by red triangles. The DRR contains a number of hypersensitive sites (HS sites) including the HS5 and HS6 enhancers [33]. (B^1–5^) In the 64YFP knock-in allele and the YAC lines Y223, DTy54 and Y001 fluorescence signal is seen in the pre-cerebellar neuroepithelium (pcn). The signal in the pcn is absent when the DRR is deleted (B^5^). (C^1–5^ ) Lateral views of dissected brains at E15.5 with anterior side to the right. GFP signal is present in the cortex (c), cerebellum (cb), optic chiasm (oc), anterior extramural streams (aes) and pontine gray (pn) nuclei, and weakly visible in the posterior extramural stream (pes) and lateral reticulate nuclei (lrn). While the cortex remains positive in the ΔDRR line, no signal is seen in the eas or pn (C^5^). (D^1–5^) Ventral views. Expression is conspicuous in the cortex (c), and olfactory bulbs (ob) of all transgenic lines. It is also clearly seen in the aes, pn and optic nerve/chiasm (oc) of the 64YFP, Y223, DTy54 and Y001 lines, but absent from the aes and pn, as well as from the optic nerve/chiasm (oc) in the ΔDRR line (E^1–3^) Dorsolateral views of the hindbrain regions with long exposure, showing signal in the precerebellar neuro-epithelium (pcn) and migratory streams (pes, aes) of the 64YFP, Y223, and DTy54 lines. Signal in the cerebella (cb) of the YAC transgenic lines is weak compared to the YFP signal of the 64YFP knock-in line. (F^1–2^) Fluorescence signal at the lateral sides of the diencephalon is seen in a transverse section through the region of an Y001 brain (F^1^), but is missing in an equivalent section through a ΔDRR brain. (G^1–5^) Skulls of E15.5 embryos after the brains have been removed. Fluorescence in a 64YFP embryo highlights Pax6 expression in the eyes and olfactory epithelium. None of the YAC transgenic lines show fluorescent signal in the olfactory epithelium (oe). Expression is present in the eyes (e) of the Y223, DTy54 and Y001 lines, but missing in the ΔDRR line, apart from expression in the cornea (white arrow in G^5^).

We have thus identified multiple cis-regulatory elements in the *Pax6* genomic locus that exhibit overlapping tissue-specific enhancer activity. Expression in the developing optic cup overlaps between the HS5 and E60A elements, the E60A and E60UCS elements share similar enhancer activity in the forebrain and the E60UCS and HS6 elements both drive expression in the precerebellar nuclei and migratory streams in the hindbrain of transgenic reporter mice (Table 2). It is hypothesized that these elements will have redundant functions in the transcriptional regulation of the *Pax6* gene. Testing this hypothesis requires the deletion of specific elements from the endogenous locus or from a large reporter transgene. We have previously described the generation of YAC reporter transgenic mice in which GFP is inserted in frame into *PAX6* in a 420 kb human YAC capable of rescuing the mouse *smalleye* mutation [23] Lines Y223 and DTy54 carry multi-copy and single copy full-length integrations respectively of the YAC, and the single-copy line Y001 lacks only the very distal end of the YAC [33], [53]. In YAC Y001 the Downstream Regulatory Region (DRR), including HS5, HS6, is flanked by LoxP sites (Figure 8A) [33]. The line Y001ΔDRR (ΔDRR) is directly derived from Y001 by Cre-mediated deletion of the DRR, and therefore has the same genomic integration site. We have compared GFP fluorescence patterns among these reporter YAC transgenic lines and to YFP fluorescence in 64YFP, a novel targeted gene-trap knock-in allele of *Pax6* (Kleinjan et al., in preparation)(Figure 8A). In the 64YFP, Y223, DTy54 and Y001 lines fluorescence is seen in the known Pax6 expression sites including cortex, eyes, optic nerves, and olfactory bulbs, as well as in the pcn, pontine migratory streams and the pre-cerebellar nuclei. Expression is readily seen in the aes and pontine grey nuclei, while signal in the pes and lrn and ecn is low and only visible upon long exposure (see Figure 8E ^1–3^ for the pes). Deletion of the DRR in line Y001ΔDRR results in a complete loss of reporter expression in the pcn, pontine migratory streams and the pre-cerebellar nuclei (Figure 8B ^5^, 8C^5^, 8D^5^), indicating that the DRR is essential for this aspect of Pax6 expression, most likely through the activity of the HS6 enhancer, as so far no other enhancers for these structures are known to reside in the DRR.

**Table 2:** Main sites of expression and overlap between the novel Pax6 enhancers.

HS5+Z	HS6Z	E60A	E60UCS
Spinal cord			
		Telencephalon (E9.5>)	Telencephalon (E10.5>)
Optic cup (Pax6 Dependent)		Optic cup (Pax6 Independent)	
Neuroretina	Neuroretina (E12.5-E16.5 only)	Neuroretina	
		Prosomere P3	
Prosomere P1 (diencephalon)		Prosomere P1 (low level)	
Lateral band around thalamus		Lateral band around thalamus (low level)	
	Rhombic lip		Rhombic lip
	Precerebellar neuroepithelium (pcn)		Precerebellar neuroepithelium (pcn)
	Posterior extramural stream (pes), lateral reticulate nuclei (lrn), external cuneate nuclei (ecn).		Posterior extramural stream (pes), lateral reticulate nuclei (lrn), external cuneate nuclei (ecn)
	Anterior extramural stream (aes), pontine gray nuclei (png)		Anterior extramural stream (aes), pontine gray nuclei (png)
		Cerebellum	
Olfactory bulbs (weak)	Olfactory bulbs (weak)	Olfactory bulbs (strong)	Olfactory bulbs (weak)
		Lateral olfactory tract (lot)	Lateral olfactory tract (lot)
		Cortical lobes	Cortical lobes

Deletion of the DRR, thus removing HS5 as well as a number of other elements from the transgenic reporter Y001, also abolishes expression in the band of expression at the lateral sides of the diencephalon (Figure 8F), suggesting that the activity of the HS5 element is essential for transcription in this structure in the embryo. Furthermore, DRR deletion abolishes expression in the optic cup, indicating that the E60A enhancer alone or with any other remaining optic cup enhancers in the locus, is insufficient to drive expression in this structure (see [33]). The lack of expression in the optic cup upon DRR deletion is reflected at later stages in the absence of GFP signal in the eyes and optic nerves of Y001ΔDRR embryos at E15.5 (Figure 8G ^5^), in comparison with the 64YFP, Y223, DTy54 and Y001 lines (Figure 8G ^1–4^).

Finally, it is notable that the YAC transgenic lines all lack expression in the olfactory epithelium (Figure 8G ^2–5^), which is positive for Pax6 expression as indicated by the 64YFP line (Figure 8G ^1^). Similarly only weak signal is seen in the cerebellae of the YAC transgenics in comparison to 64YFP (Figure 8G ^1–3^). As Y223, DTy54 and Y001 are independent lines this suggests that essential enhancers for expression in these tissues are missing from the reporter YAC.

In summary, we have characterised three novel enhancer regions in the *Pax6* locus, identified through a combination of sequence conservation and DNaseI hypersensitivity mapping. These enhancers drive distinct but partially overlapping tissue-specific expression patterns, and depend on the presence of each other to regulate transcription in the genomic *Pax6* locus. The complex cis-regulatory landscape produced by these elements in combination with the many previously characterised *Pax6* enhancers most likely reflects the highly specific requirements for strict spatio-temporal and quantitative control of *Pax6* gene product during development.

## Discussion

The generation of complex vertebrate body plans and the formation of specialised organs critically depend on the controlled expression of a set of developmental regulator genes, mostly comprising transcription factors and signaling molecules. These developmental control genes generally act as part of specialised gene regulatory networks (GRN), and the same gene may be part of many separate GRNs [54]. PAX6 is a transcription factor that has been co-opted into several GRNs that are key to the correct development of the eye, brain, pancreas and olfactory structures. In many tissues PAX6 has an essential role at multiple stages of development, e.g. in lens formation early PAX6 expression in the surface ectoderm is essential for formation of the lens placode [12], while later expression in the lens vesicle is necessary for lens fiber differentiation and crystallin gene expression [55]. An appropriate level of expression is also important, as both overexpression and haplo-insufficiency of Pax6 are known to have phenotypic consequences for eye and brain development [21]–[26]. It is therefore unsurprising that transcription of the *Pax6* gene is controlled through the existence of a large number of cis-regulatory elements. A more complete knowledge of the *Pax6* cis-regulatory landscape is necessary for understanding of transcriptional mechanisms and controls, and to appreciate how and where these may be disrupted in disease, including phenotypes other than aniridia.

In the past evolutionary sequence conservation has been used successfully to identify novel *Pax6* cis-regulatory elements. To complement and compare with this strategy we have here used a different, unbiased approach to analyse the chromatin at the mouse *Pax6* locus in three murine cell lines of different origins, MV+, a lens epithelium derived cell line, N2A, neuroblastoma, and RAG, kidney carcinoma. We found an unexpectedly large number of hypersensitive sites, in particular in the MV+ cell line, indicating complex chromatin architecture at the locus in these cell lines. The identified HS sites can be grouped into tissue-specific HS sites and general HS sites. Nearly all general HS sites, i.e. those found in all three cell lines, occur at interspersed repeats. Very little is known about the role of repetitive elements in gene regulation, but some repeats are suggested to influence transcription at nearby genes. Many of the tissue-specific hypersensitive sites map to sequences that are highly conserved over a long evolutionary time-scale, but some identify elements that are less deeply conserved. Tissue-specific nuclease sensitive regions are often correlated with regulatory elements, such as enhancers, repressors or insulators [30], [40], [41], [47]. We selected three fragments of varying DNase hypersensitivity, and level and depth of sequence conservation, for functional characterization in reporter transgenic mice.

The first fragment analysed, HS5+, contains two deeply conserved sequence elements, with conservation easily recognizable in frog, chicken and fish. However, while one of the sites was previously identified as an HS site in the human lens cell line CD5a [30], neither of the two conserved elements displayed DNaseI hypersensitivity in the three mouse cell lines used here. This could indicate a species difference between mouse and human, but is more likely to be due to cell type differences between CD5a and MV+, despite the lens epithelial origin and similar derivation of both cell lines. This is further underscored by the observation of clear hypersensitivity at the SIMO lens enhancer in MV+ cells in this study whereas no obvious HS site was detected in CD5a cells [30].

Characterisation of the HS5+ fragment revealed tissue-specific enhancer function driving reporter gene expression in the developing optic cup and neuroretina, in the spinal cord and in the prosomere P1 region of the diencephalon. These expression sites are a subset of the wider Pax6 expression domain and multiple other enhancers have already been described for these expression sites [28]–[33], emphasizing the complexity of the *Pax6* regulatory landscape. Since autoregulation is a known feature of Pax6 expression [7], [24], [32], [33] we analysed reporter expression driven by the element in a Pax6 null background (i.e. in *Pax6* ^sey/sey^ mice [20], [21]. This demonstrates that the modules within the cis-element that drive expression in diencephalon and eye are dependent on Pax6 while the spinal cord module of the enhancer works independently of Pax6. Autoregulation is a frequent feature of genes with a role in lineage maintenance [56]. Dependence of the HS5 regulatory element on Pax6 expression suggests the role of this element is to maintain expression in the optic cup and diencephalon, but that initiation of expression at these tissues is governed by another cis-element(s) in the locus. Nevertheless, deletion of the wider DRR region, including HS5 and several additional cis-elements, from a reporter YAC transgenic model resulted in a complete lack of reporter expression in the optic cup and neuroretina, suggesting the initiating cis-element resides within the DRR region [33]. As not all HS sites and conserved elements within the DRR have yet been characterized it remains possible that other early eye specific enhancers are present in the region.

Hypersensitive site 6 (HS6) is a strong tissue-specific HS site within the DRR region that is found in both the mouse MV+ lens cells and the human CD5a lens cells [30]. Unlike many of the HS sites, HS6 is a cis-element without deep sequence conservation: sequence homology is confined to mammalian species (including marsupials and monotremes (Figure S6)), but not detectably found in chicken, xenopus or fish. Hence without the HS site mapping the sequence would likely have escaped recognition as a cis-regulatory element. The HS6 element drives reporter expression in the developing eye during a restricted time period and in the precerebellar neuron-epithelium of the hindbrain and its derived precerebellar nuclei. It remains to be further investigated whether the element constitutes a newly evolved regulatory sequence in mammalian lineages only, or whether the element is more ancient, but has been lost in all other lineages. The alternative possibility that the primary sequence has diverged but function has been preserved, as reported for some other cases [39], [57] is unlikely considering the high level of conservation of other elements in the *Pax6* locus. The very distinct expression pattern of the HS6-LacZ reporter in the pcn, migratory streams and pre-cerebellar nuclei make it a useful tool for the analysis of specific mouse mutants and the design of screens for the detection of new genes involved in the development and function of these structures. To demonstrate this we have used the HS6Z transgene as a marker to highlight known migratory defects of the posterior extramural stream (pes) in *Pax6* ^sey/sey^ embryos. Consistent with a recent report using an ex-vivo system [50], analysis of HS6Z/ *Pax6* ^sey/sey^ embryos shows a delay of the pes at the midline, a disorganized structure of the lateral reticulate nucleus and absence of the external cuneate nucleus. In addition we show here that migration along the anterior extramural stream is also reduced, with concomitant absence of the ponto-cerebellar tract, and that a subset of cells follow an aberrant migratory path to settle in an ectopic position in the pons. The functional consequences of the misdevelopment of these structures in the absence of *Pax6* are presently unknown as *Pax6* ^sey/sey^ mice die at birth [20], [21]. The specificity of the HS6 enhancer should make it possible to engineer mice lacking Pax6 function only in the pcn and derived structures, and thus allow analysis of adult mice assuming they are viable.

Although identification of an HS site reliably indicates the presence of a functional cis-element in those cells, tissue specificity is not easily predicted from the nature of the immortalised cell lines in which it was found. Characterisation of HS sites in transgenic reporter mice in this and previous studies indicates that most of the MV+ specific HS sites do express in the eye, albeit in a variety of stages and cell types, suggesting the MV+ cell line represents a more general characteristic of eye tissue rather than being strictly lens epithelium specific.

Recently a new report described a Turkish family in which several family members have aniridia [58]. Affected family members were found to carry a large deletion downstream of *PAX6*, but not including the *PAX6* coding region. The proximal breakpoint of the deletion was mapped to a position located beyond the previously most distal mapping aniridia patient breakpoint (SIMO; [34], [35], [30]), leaving the SIMO lens and HS2/3 retinal enhancers intact and in cis with *PAX6*, but removes the HS5 and HS6 elements described here, as well as any further downstream cis-elements [58]. It is unclear whether the aniridia phenotype in the Turkish family is milder than that found in other patients, but it strongly suggests that the elements identified here, and/or other as yet unidentified distal enhancers, are essential to drive proper levels of Pax6 in the developing eye.

The use of multi-species sequence conservation has proved a successful strategy for the identification of *Pax6* cis-regulatory elements [32], [33]. Among the many highly conserved sequences around *Pax6* one element stands out for its extreme level of sequence conservation. This element, termed E60UCS, is one of the longest elements among the class of 480 ultra conserved sequences (UCS) in the human genome, defined as elements that exhibit 100% sequence conservation over 200bp or more between human, mouse and rat [51]. Although no specific DNase hypersensitive site was found in our cell lines, there appeared to be an increased general DNase sensitivity over the E60 region compared to other non-HS site containing fragments in the locus, suggestive of a wider open chromatin structure in this region. Further assays will be necessary to confirm this observation. Transgenic reporter assays of the E60UCS and adjacent E60A elements revealed the region to have enhancer activity in a wide ranging pattern, including in sites that overlap with the expression patterns of the HS5 and HS6 enhancers, suggesting they might act in a cooperative or hierarchical manner. To characterise the interdependency of the enhancers we used our panel of GFP reporter YAC transgenic lines to show a loss of expression in regions of overlap between the HS5+, HS6 and E60 enhancer regions in the absence of the DRR. This demonstrates that the E60 enhancer, which remained present with the rest of the reporter locus, is insufficient by itself to drive expression in these tissues in a genomic, single copy context.

Two recent studies have reported the presence of two enhancers with overlapping expression patterns near the *snail* and *shavenbaby* genes in Drosophila, one near the gene and a second at a more distal position [59], [60]. The more distally located enhancer was termed ‘shadow enhancer’ in these reports, even though both enhancers were shown to behave similarly with respect to sufficiency and necessity. Deletion of either enhancer in those cases did not result in any obvious phenotype or effect on reporter expression, and only in a sensitized genetic background or when environmentally challenged was an effect observed, giving rise to the conclusion that the role of ‘shadow enhancers’ is to provide robustness through the buffering provided by the two equivalent enhancers. Our data showing overlapping expression domains driven by multiple individual cis-elements in the *Pax6* locus initially suggests a similar situation. However, as our reporter YAC lines demonstrate, here the enhancers are clearly not redundant, as the loss of one enhancer (albeit in the context of the larger DRR deletion) results in complete loss of expression despite the presence of the second (and possibly other additional) enhancers. It is conceivable that the existence of functionally non-equivalent enhancers with overlapping expression domains has evolved from an initial situation of true ‘shadow enhancers’, as still seen in Drosophila. Such a situation could then allow for more precise fine-tuning of the expression. The regulatory architecture at the mammalian *Pax6* locus fits with a cis-regulatory logic of initiating and maintenance elements, where the maintenance enhancer is dependent on expression of the target gene through an auto-regulatory feedback loop, while the initiating enhancer for obvious reason has to be independent of its own expression. While such a scenario fits our observations regarding expression in the pre-cerebellar neuro-epithelium and derived structures, the situation appears more complex for the optic cup. Although expression driven by the HS5+ element in the optic cup depends on Pax6 and the E60A element does not, loss of the DRR including HS5+ results in complete loss of optic cup reporter expression despite the presence of E60. This suggests that another, as yet unidentified enhancer located in the DRR, is the primary element for optic cup expression of Pax6. This demonstrates the need to chart the complete regulatory landscape around developmental regulatory genes in order to unravel the intricacies of their regulated expression, and to provide insight into which regulatory defects could lead to disease.

In conclusion we show that a combined approach of using DNase HS site mapping and multi-species sequence conservation has led to the identification of several novel enhancer elements in the *Pax6* locus. Detailed characterisation of the expression patterns elicited by these enhancers reveals overlapping and distinct functions. It has not escaped our attention that the analysis of cis-acting sequences with overlapping expression patterns could provide a way to unlocking the TF binding site code required for expression in the specific tissues. Remarkably, considering the strong overlap in expression patterns, we were unable to find extended stretches of sequence similarity between the separate elements, suggesting the elements are not directly derived from each other through a relatively recent duplication event. Future studies using more sophisticated bio-informatic approaches may be able to make use of the overlap in expression patterns to deduce candidate upstream regulators of these cis-elements. The presence of multiple enhancers for the various overlapping expression domains of Pax6, dependent on different sets of upstream acting transcription factors, may allow for a more precise fine-tuned control over the functional output of this highly dosage sensitive gene.

## Materials and Methods

### Ethics Statement

All animal experiments were approved by the University of Edinburgh ethical committee (approval ID TR-15-08) and performed under UK Home Office license number PPL 60/3785.

### Cell lines, RNA extraction and rtPCR

The MV^+^ lens cell line derived from the lens epithelia of a wild type C57Bl6 littermate control of the Vimentin knock-out mouse [44]. These cells were a gift from Dr Alan Prescott (Dundee). The renal adenocarcinoma (RAG) kidney cell line [46] was derived from BALB/c mice and obtained from the American Type Culture Collection (ATCC; http://www.lgcpromochem-atcc.com/). The Neuro2a (N2A) cell line (N2A) [45] was derived from a neuroblastoma of an albino mouse and is also available from the ATCC. These cells were a gift from Dr David Price (Edinburgh).

RNA was isolated from the cell lines using TRI reagent (Sigma) according to manufacturer's protocol. RT-PCR was performed using AMV reverse transcriptase and random hexamers for first strand synthesis. Specific PCR primers used were:

Pr049: 5′-TAGATGGGCGCAGACGGCATG-3′

Pr050: 5′-AGATCTATTTTGGCTGCTAGTC-3′;

The ubiquitously expressed Ercc3 gene was used as control for the RNA isolation and reverse transcriptase reaction. Primers used were:

Ercc3_F: 5′-GAGGTGCACACCATTCCAG-3′

Ercc3_R: 5′-TCGGTAGAACTCGGGAGACA-3′

### DNase I Hypersensitive Site (HS) mapping

DNase I treatments were carried out as described previously [30], using approximately 5x10^8^ nuclei per experiment. Cells were harvested and washed twice in 50 ml Phosphate Buffered Saline (PBS), before re-suspending in 2 ml buffer A (10 mM HEPES pH 7.9, 10 mM KCl, 0.1 mM EDTA, 0.1 mM EGTA, 1 mM DTT) and incubated for 15 min on ice. Cells were passed through a 0.80×40 mm needle to obtain a single cell suspension, then 40 µl 10% NP-40 (0.002% final volume) was added to disrupt the cell membrane. Successful nuclei preparation was assessed by Trypan Blue staining. Nuclei were collected by centrifugation at 1500 r.c.f. for 30 seconds and resuspended in HSS buffer (15 mM Tris.HCl pH 7.4; 60 mM KCl; 15 mM NaCl; 0.2 mM EDTA; 0.2 mM EGTA; 5% glycerol; 1 mM DTT; 0.15 mM Spermine; 0.5 mM Spermidine) in 10 equal aliquots containing approximately 5x10^7^ nuclei each, which were incubated with 0, 100, 200, 300, 400, 600, 800, 1000, 2000 or 3000 Units of DNase I (Roche) and 50 µl 100 mM Mg Cl_2_ for 25 min on ice. To maintain a single nuclei suspension the reactions were mixed by gentle flicking at regular intervals. The reaction was stopped by addition of 20 µl 0.5 M EDTA (the sample incubated with 0 units of DNase I had EDTA added immediately to prevent cleavage by endogenous DNases). Nuclei were dissociated overnight by addition of 25 µl 20% SDS and 100 µl Proteinase K (10 mg / ml) to each reaction at 37°C. DNA was recovered by phenol / chloroform extraction followed by ethanol precipitation. Recovered DNA was treated with 5 µl RNase (10 mg/ml) for 30 minutes at 37°C. Successful DNase I treatment was confirmed by gel electrophoresis of 10% of each reaction. 20 µg aliquots of DNase I treated DNA were incubated overnight with an appropriate restriction enzyme and run on a 1% agarose gel. A 0.2 µg λ DNA / *Hin*d III marker was run alongside to assist in the determination of fragment size after radioactive hybridisation. Size separated DNA was transferred to Zeta-probe^®^ Genomic Tested membrane (Biorad) overnight by capillary blotting according to standard procedure. The membrane was baked at 80°C for 2 hours. Probes were generated by PCR, designed to avoid repetitive sequences, and subsequently sequenced to confirm their correct identity.

### ChIP method

ChIP was performed as previously described [61] with some minor modifications outlined below. Briefly, cells were crosslinked in a final concentration of 1% formaldhyde for 10minutes at room temperature, before quenching with 2M glycine. Cells were washed and harvested in cold PBS, chromatin was isolated and sonicated on ice through 11 rounds of sonication (30 seconds on, 30second off) with a Soniprep 150 probe sonicator. Sonicated chromatin was snap frozen and stored at -80°C. 100 µg sonicated chromatin was used for each ChIP, with a 10% input sample retained for qPCR quantification. Precleared chromatin was incubated overnight at 4°C with 25 µl Protein G Dynabead (Invitrogen) preloaded with antibody, either 2 µg Anti-trimethyl-Histone H3 Lys4 (07-473 Upstate) or control Rabbit IgG (I5006 Sigma). Beads where washed in RIPA buffer, chromatin eluted from beads, crosslinks reversed, and DNA purified. Two separate experimental replicates of each ChIP were performed on each of the three cell lines and quantified using Q-PCR.

### Quantitative Real-Time PCR

Real time PCR using Sybr Green chemistry was used to quantify ChIP samples, with each reaction performed in duplicate. Primers where designed with Primer3 to specific sites in the *Pax6* promoter regions plus controls and validated for efficiency with standard curves and melting curves. Q-PCR was performed on the LightCycler® 480 System (Roche), and Cp values were used with percentage input method to calculate relative enrichment at each primer region. Sequences of primers used are shown in Table S1.

### Comparative sequence analysis

PIP plots were made using the programme PIPmaker (http://bio.cse.psu.edu/pipmaker/) [42]. Evolutionary sequence comparison of element E60 was prepared using the VISTA program [62] with a window size of 50 bp and a minimal sequence identity of 70% using sequences derived from Z83001 (human), AL512589 (mouse), AL021531 (pufferfish), and AL929172, BX004784 (zebrafish *Pax6*a), AC127461, BX000453 (zebrafish *Pax6*b). Xenopus sequence was obtained from *Xenopus tropicalis* genome assembly 3.0 from the JGI.

The sequence line-up of the mammalian specific HS6 element was made using sequences retrieved from Ensembl release 64 (http://emsembl.org/). The following sequences were aligned: human (homo sapiens chr11:31667250-31667755), pig (sus scrofa chr2:27091000-27092000), dolphin (tursiops truncates scaffold 2669:256000-256500), cow (bos Taurus chr15:63212200-63213000), shrew (sorex araneus scaffold 1709; 112000-112700), mouse (mus musculus chr2: 105636000-105636700), horse (equus caballus chr7: 97398500-97399500), squirrel (spermophilus tridecemlineatus scaffold 5397: 323000-324000), possum (monodelphis domestica (chr5: 267244400-267244900), wallaby (macropus eugenii scaffold 83403: 200–900) and platypus (ornithorhynchus anatinus chr3: 17772000-17773000).

### Production of LacZ reporter constructs

The E60+Z, E60A-Z and E60UCS-Z reporter constructs are made using a modified p610 vector containing a hsp68-*LacZ* reporter cassette (p610+, [30]). A 2.3 kb fragment containing the E60+ conserved region (hg19 assembly chr11:31,784,564-31,786,850) was PCR amplified from human cosmid G0453 (EMBL Z83001) using high fidelity polymerase (Bio-X-Act) with primer pair 5′-TGTCGACCAATGCAGCACCAAAGTGTATGC-3′ and 5′-AGTCGACAAAAGATAAGTAAGCTCAGATGT-3′, and cloned into the SalI site of the p610+ vector to generate E60Z. The E60A-Z and E60UCS-Z microinjection fragments were generated using the Asp718I restriction site located between the two highly conserved subregions of the full-length E60+ fragment.

The HS5+-Z construct was made by PCR amplification of a 3.4 kb fragment (hg19 assembly chr11:31,669,799-31,673,207) with primer pair: 5′-TGCTTTCCTTGTGACACT-3′ and 5′-TTACAGATTCCTAGTCAGT-3′, cloned into pGEM-T (Promega), followed by subcloning into the SphI and SalI sites of the p610+ LacZ reporter vector using a partial SphI/SalI digest.

The HS6-Z construct was made by PCR amplification of a 1.8 kb fragment (hg19 assembly chr11:31,667,071-31,668,901) using primer pair 5′-AGTCGACAGCAGGATTCAGAAGCA-3′ and 5′-CATATAATTGAGAAAGTCCTAGT-3′, cloned into pGEM-T, and subcloned into the SalI site of p610+.

### Production and analysis of reporter transgenic mice

Microinjection fragments were generated by digestion with suitable restriction enzymes, isolated from agarose gel using Qiagen gel extraction columns and microinjected according to standard procedures. Transgenic mice and embryos were identified by PCR using LacZ and element specific primers. Embryos were collected at the appropriate stages, washed in PBS and fixed for 1 hour in a solution of 1% formaldehyde; 0.2% glutaraldehyde; 2mM MgCl_2_; 5mM EGTA and 0.02% NP-40 in PBS. After fixation the embryos were washed in PBS containing 0.02% NP-40, before being stained for several hours at 37^o^C in the dark in a solution containing 5mM K_3_Fe(CN)_6_; 5mM K_4_Fe(CN)_6_.3H_2_O; 2mM MgCl_2_; 0.01% sodium deoxycholate; 0.02% NP-40 and 0.1% 5-bromo-4-chloro-3-indolyl-β-D-galactopyranoside (X-gal). Embryos were photographed on a Leica MZ FLIII Microscope fitted with a Hamamatsu Orca-ER digital camera with a CRI micro-color filter.

Production of the GFP YAC reporter transgenic mice has been described [33].

## Supporting Information

Figure S1 **DNase I hypersensitive site mapping across the mouse Pax6 locus.** Sequences from the mouse Pax6 genomic region from 20 kb upstream of the Pax6 P0 promoter to approximately 170 kb downstream of the gene (mm9/NCBI37 position chr2:105,495,760 to chr2:105,678,886) were examined for presence of DNase I HSs by Southern blot (figures S1, S2, S3, S4, S5: The thick line in the schematic overview of the Pax6 locus at the top of the figure indicates the total region analysed. Pax6 exons are indicated by black boxes above the line, exons of the adjacent Elp4 gene, transcribed in opposite direction are below the line. Selected enhancers are shown as black or orange ellipses, the Pax6 downstream regulatory region (DRR) is shown with red downward arrows). Figure S1 shows the analysis of A) the 0–20kb region and B) the 20–40kb region. Blots inside each coloured box represent analysis of one restriction fragment and blots for the three cell lines used are grouped (columns left to right: MV+, N2A, RAG). Coloured boxes are labelled at the top left hand corner with the name of the restriction enzyme used to generate the (parent) fragment with the size of the fragment in kilobases. The parent fragment is indicated to the left of the blots by a coloured line representing the probe used (with its name in white text), corresponding to the coloured fragment shown below the PIP plot. Hypersensitive degradation fragments are labelled in black with their approximate size in kb in brackets. Marker and DNase I lanes are indicated at the top of each column; triangle represents increasing DNase I enzyme concentration; 0 =  no DNase I control. The Pax6 locus is represented in 10 sets of 20 kb segments by a percentage identity plot (PIP) rotated 90° clockwise and positioned to the right of the HS mapping blots. The x axis represents the mouse genomic sequence which is compared to sequences from the human and chicken genomes (upper and lower y axis) in 100 bp windows. Sequence identity greater than 50% in the 100 bp window between the compared sequences is plotted on the appropriate y axis. Exons are indicated by pink shaded boxes on the PIP output, Pax6 introns are shaded in yellow, Elp4 introns in orange. Additional genomic features recognised by the PIP maker program are represented above the PIP and correspond to various repetitive elements and CpG islands (see http://pipmaker.bx.psu.edu/pipmaker/ for further details). Probes and restriction fragments used in DNase I HS analysis are colour-coordinated. Restriction fragments are drawn in tracks below the PIP, with Southern probes shown below these tracks. DNase I HSs are summarised above the PIP with data from each cell line presented in a separate track using a black or grey line: MV+ - top, N2A - middle, RAG - bottom.
(TIF)

Figure S2 **DNase I hypersensitive site mapping across the Pax6 genomic locus, part 2, covering C) the 40**–**60 kb segment and D) the 60**–**80kb segment of the locus as indicated on the map of the locus at the top of the figure.** Full details are given in the legend for figure S1.
(TIF)

Figure S3 **DNase I hypersensitive site mapping across the Pax6 genomic locus, part 3, covering E) the 80**–**100 kb segment and F) the 100**–**120kb segment of the locus as indicated on the map of the locus at the top of the figure.** Full details are given in the legend for figure S1.
(TIF)

Figure S4 **DNase I hypersensitive site mapping across the Pax6 genomic locus, part 4, covering G) the 120**–**140 kb segment and H) the 140**–**160kb segment of the locus as indicated on the map of the locus at the top of the figure.** Full details are given in the legend for figure S1.
(TIF)

Figure S5 **DNase I hypersensitive site mapping across the Pax6 genomic locus, part 5, covering I) the 160**–**180 kb segment and D) the 180**–**200kb segment of the locus as indicated on the map of the locus at the top of the figure.** Full details are given in the legend for figure S1.
(TIF)

Figure S6 **Sequence line-up for the HS6 element.** The HS6 element is conserved among mammalian species only, with platypus and wallaby being the evolutionarily most distant species with the conserved element. No conservation was detected to non-mammalian genomes.
(DOC)

Table S1 **Primer sequences and genomic positions of Q-PCR primers used for H3K4me3 Chromatin Immunoprecipitation.**
(DOC)
