# Supplementary figures and images for: DNaseI Hypersensitivity and Ultraconservation Reveal Novel, Interdependent Long-Range Enhancers at the Complex Pax6 Cis-Regulatory Region
Source: PLoS One. 2011 Dec 29;6(12):e28616. doi: 10.1371/journal.pone.0028616 (PMC3248410; doi:10.1371/journal.pone.0028616)

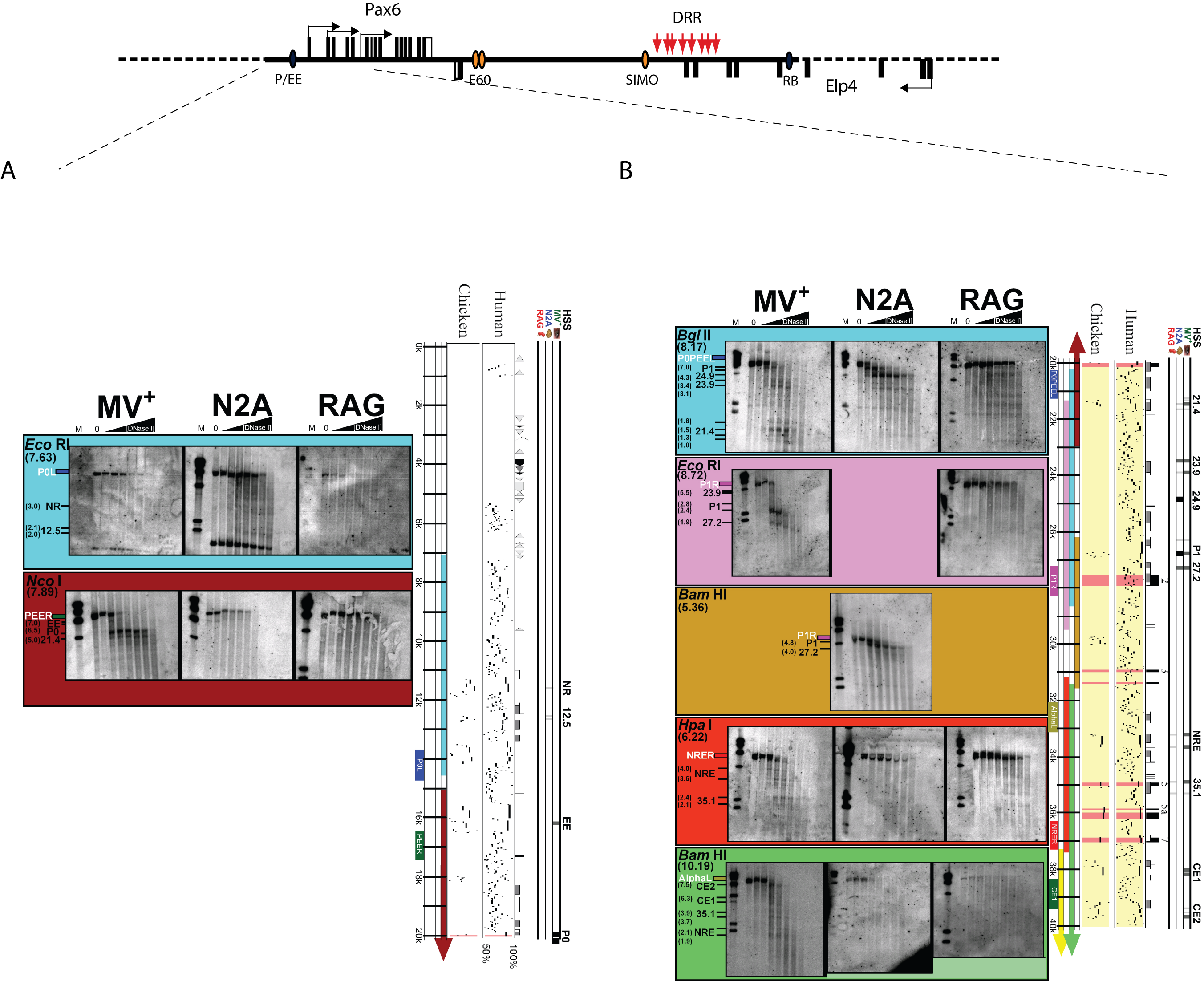

Supplement: Figure S1 — DNase I hypersensitive site mapping across the mouse Pax6 locus. Sequences from the mouse Pax6 genomic region from 20 kb upstream of the Pax6 P0 promoter to approximately 170 kb downstream of the gene (mm9/NCBI37 position chr2:105,495,760 to chr2:105,678,886) were examined for presence of DNase I HSs by Southern blot (figures S1, S2, S3, S4, S5: The thick line in the schematic overview of the Pax6 locus at the top of the figure indicates the total region analysed. Pax6 exons are indicated by black boxes above the line, exons of the adjacent Elp4 gene, transcribed in opposite direction are below the line. Selected enhancers are shown as black or orange ellipses, the Pax6 downstream regulatory region (DRR) is shown with red downward arrows). Figure S1 shows the analysis of A) the 0–20kb region and B) the 20–40kb region. Blots inside each coloured box represent analysis of one restriction fragment and blots for the three cell lines used are grouped (columns left to right: MV+, N2A, RAG). Coloured boxes are labelled at the top left hand corner with the name of the restriction enzyme used to generate the (parent) fragment with the size of the fragment in kilobases. The parent fragment is indicated to the left of the blots by a coloured line representing the probe used (with its name in white text), corresponding to the coloured fragment shown below the PIP plot. Hypersensitive degradation fragments are labelled in black with their approximate size in kb in brackets. Marker and DNase I lanes are indicated at the top of each column; triangle represents increasing DNase I enzyme concentration; 0 = no DNase I control. The Pax6 locus is represented in 10 sets of 20 kb segments by a percentage identity plot (PIP) rotated 90° clockwise and positioned to the right of the HS mapping blots. The x axis represents the mouse genomic sequence which is compared to sequences from the human and chicken genomes (upper and lower y axis) in 100 bp windows. Sequence identity greater than 50% [file pone.0028616.s001.tif]

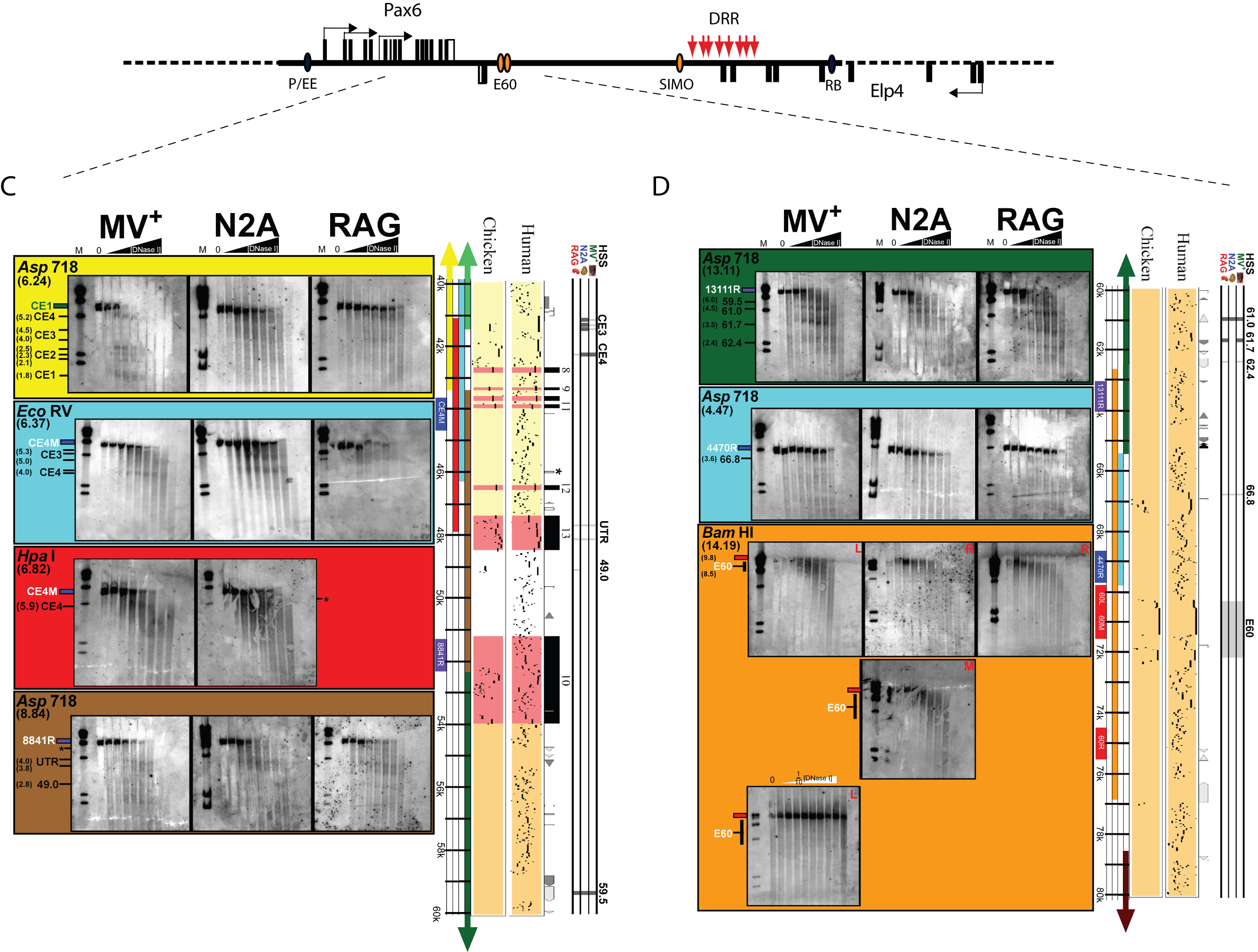

Supplement: Figure S2 — DNase I hypersensitive site mapping across the Pax6 genomic locus, part 2, covering C) the 40–60 kb segment and D) the 60–80kb segment of the locus as indicated on the map of the locus at the top of the figure. Full details are given in the legend for figure S1. (TIF) [file pone.0028616.s002.tif]

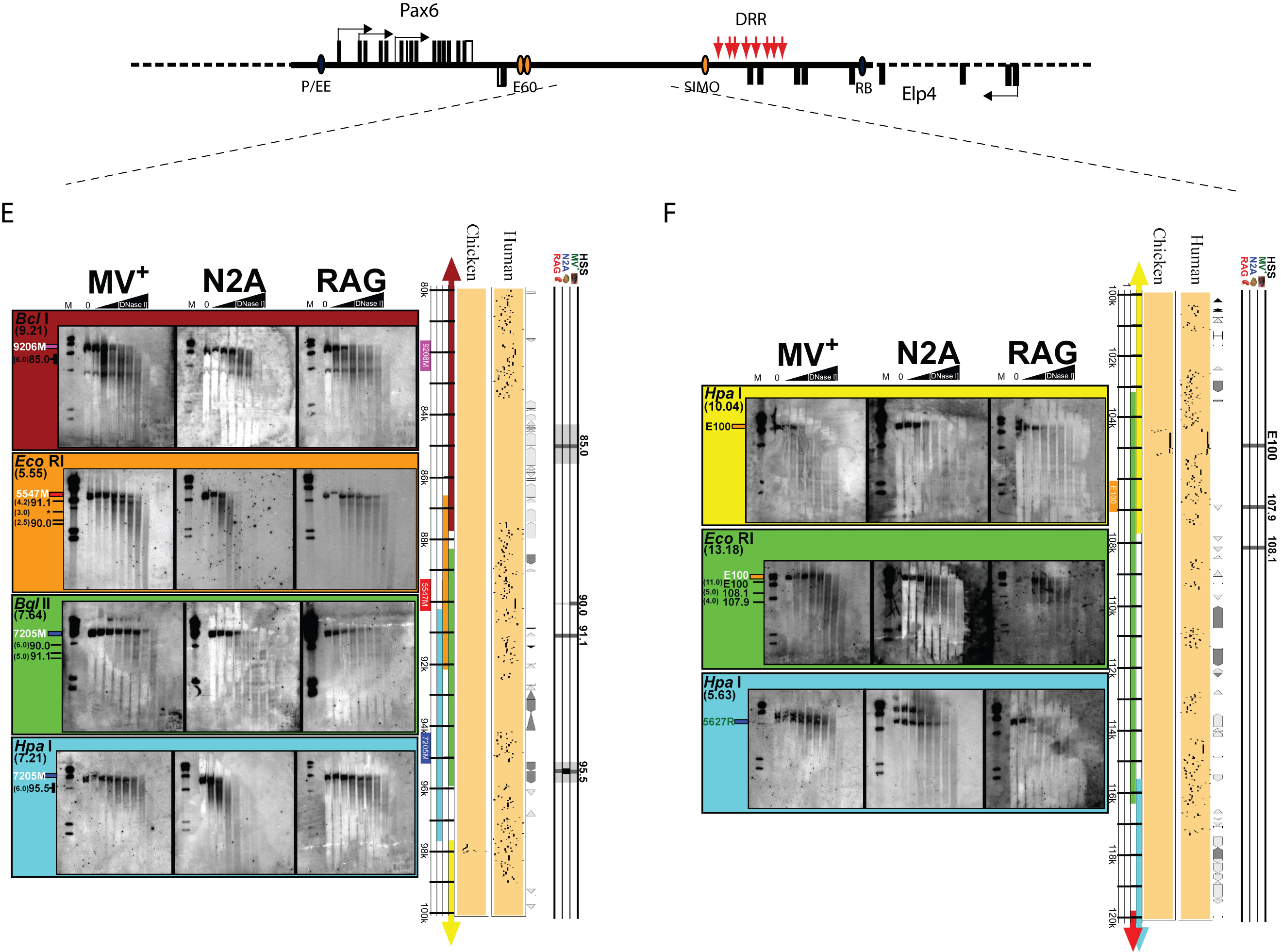

Supplement: Figure S3 — DNase I hypersensitive site mapping across the Pax6 genomic locus, part 3, covering E) the 80–100 kb segment and F) the 100–120kb segment of the locus as indicated on the map of the locus at the top of the figure. Full details are given in the legend for figure S1. (TIF) [file pone.0028616.s003.tif]

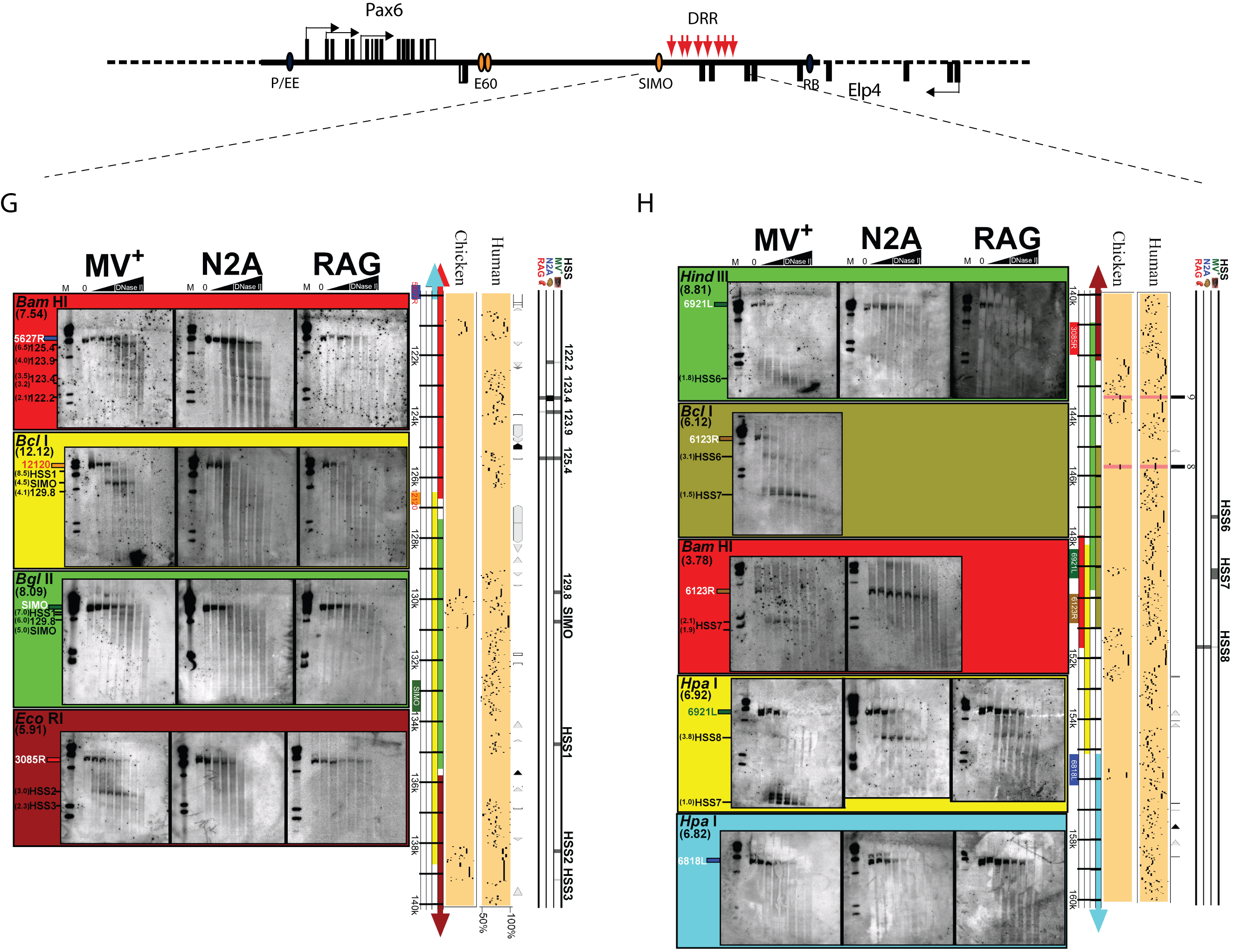

Supplement: Figure S4 — DNase I hypersensitive site mapping across the Pax6 genomic locus, part 4, covering G) the 120–140 kb segment and H) the 140–160kb segment of the locus as indicated on the map of the locus at the top of the figure. Full details are given in the legend for figure S1. (TIF) [file pone.0028616.s004.tif]

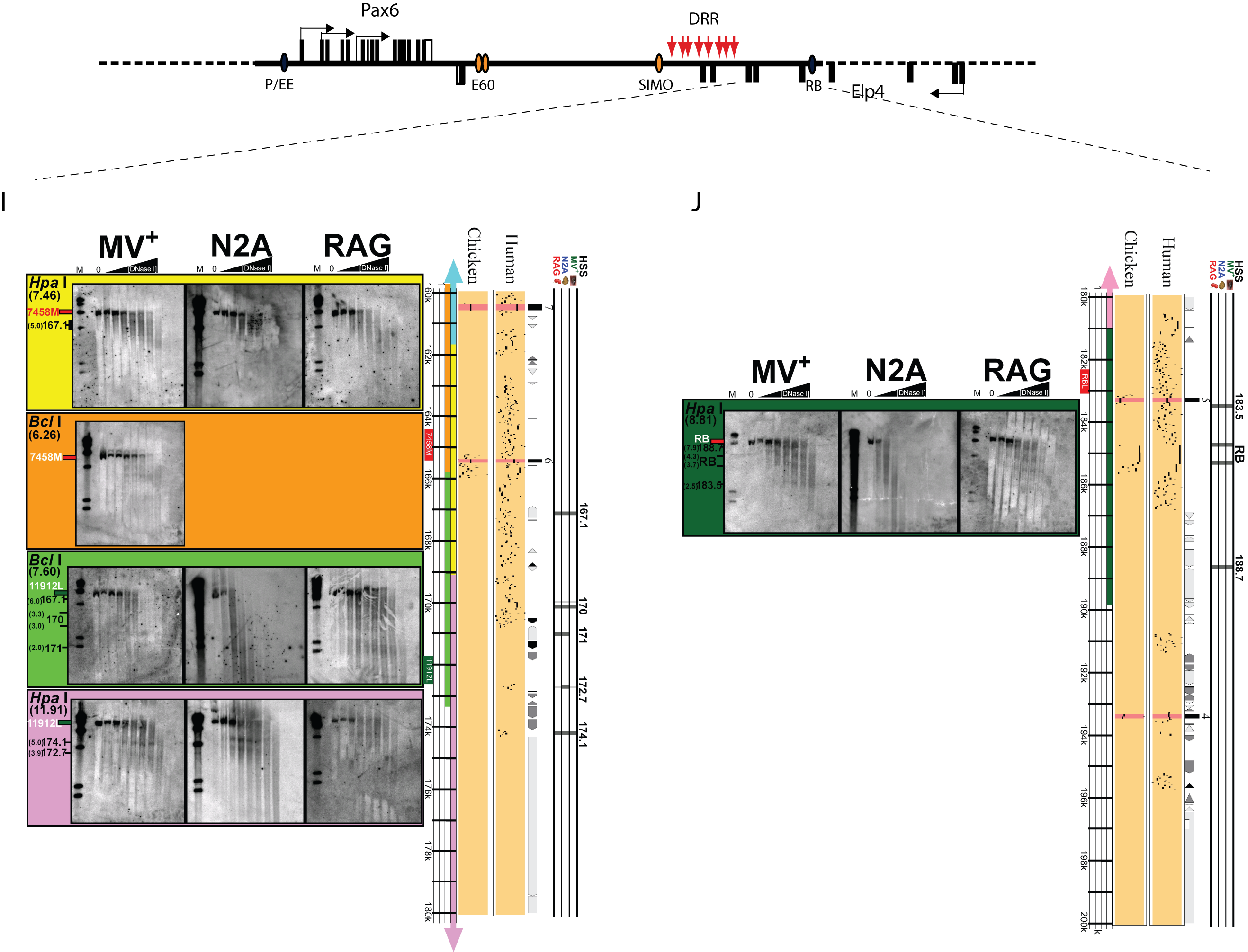

Supplement: Figure S5 — DNase I hypersensitive site mapping across the Pax6 genomic locus, part 5, covering I) the 160–180 kb segment and D) the 180–200kb segment of the locus as indicated on the map of the locus at the top of the figure. Full details are given in the legend for figure S1. (TIF) [file pone.0028616.s005.tif]

Supplementary figure S6:


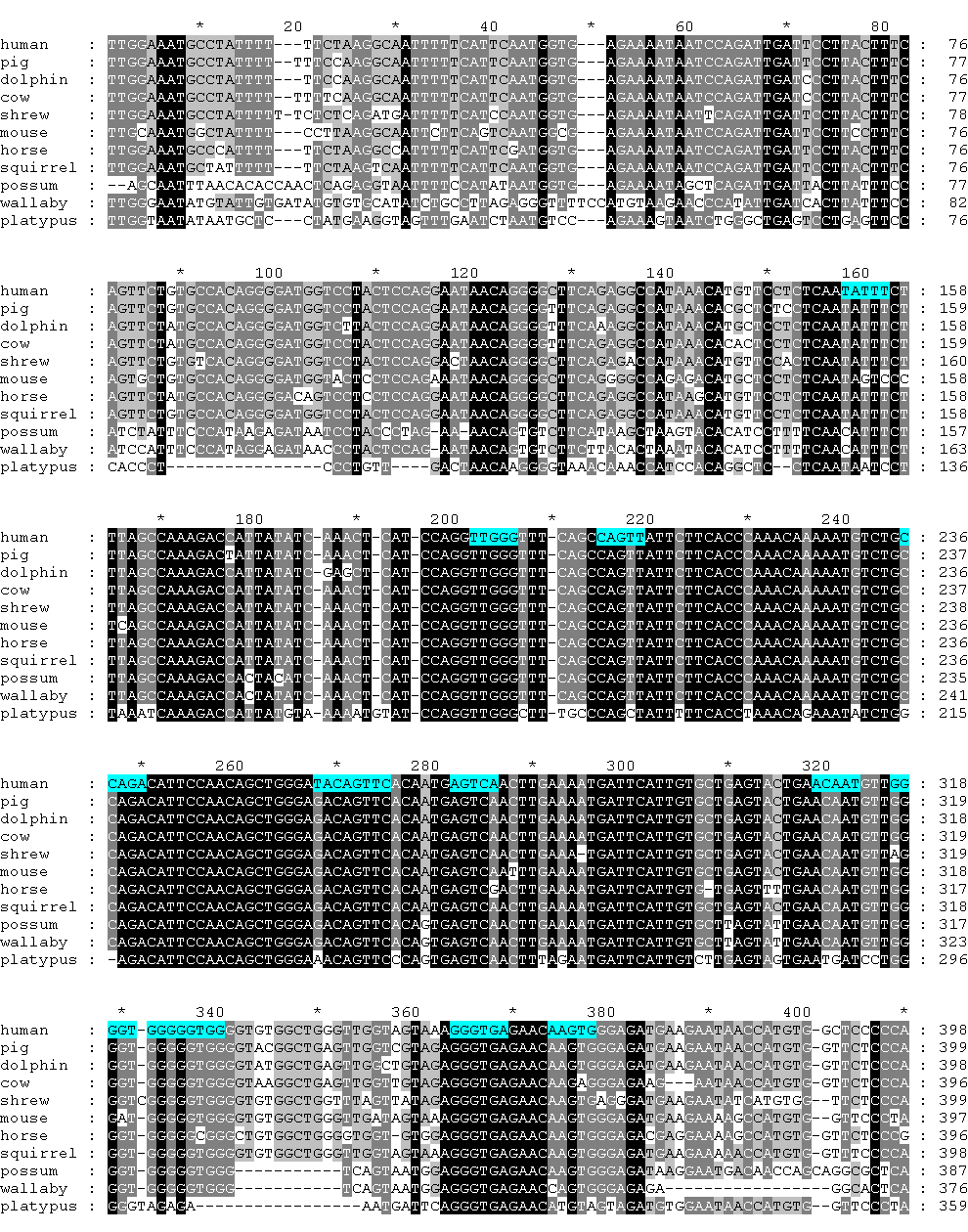


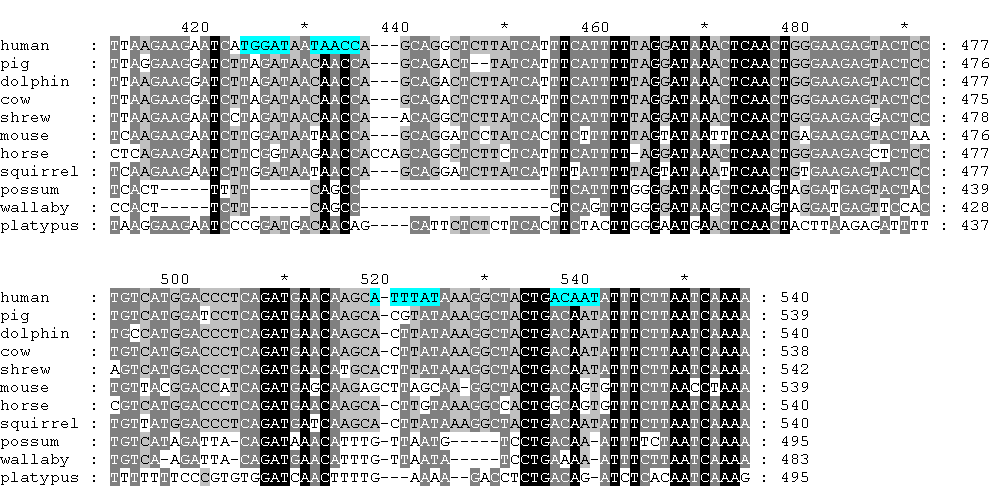

Supplement: Figure S6 — Sequence line-up for the HS6 element. The HS6 element is conserved among mammalian species only, with platypus and wallaby being the evolutionarily most distant species with the conserved element. No conservation was detected to non-mammalian genomes. (DOC) [file pone.0028616.s006.doc]
