# Supplementary material for: DNaseI Hypersensitivity and Ultraconservation Reveal Novel, Interdependent Long-Range Enhancers at the Complex Pax6 Cis-Regulatory Region
Source: PLoS One. 2011 Dec 29;6(12):e28616. doi: 10.1371/journal.pone.0028616 (PMC3248410; doi:10.1371/journal.pone.0028616)
Supplement: Table S1 — Primer sequences and genomic positions of Q-PCR primers used for H3K4me3 Chromatin Immunoprecipitation. (DOC) [file pone.0028616.s007.doc]

| Name | Forward primer | Reverse primer | Genomic coordinates NCBIM37 |
| --- | --- | --- | --- |
| Pax6.P0. - 5kb | CCTAGACCTGCCCTGTTCTG | CCTGCCCAAATCTAGGATGA | chr2:105503585-105503698 |
| Pax6.P0. - 2.5kb | TGACCTGCAAGAAGACACAGA | CTAGCATTCCAGGGTGGGTA | chr2:105505988-105506116 |
| Pax6.P0. Promoter | GGAGGACAATACCAGCCAGA | GGTTCAGCTCGGCAGATTAG | chr2:105508818-105508961 |
| Pax6.P1. - 2.5kb | ATTTGTTTGCCATCCCAGAG | AGGAGGGCAAGGATGAATTT | chr2:105512283-105512418 |
| Pax6.P1. - 1.3kb | GGCTGATTGAAAGAGCCAGT | GCAACCCAGTGGATTAGCAC | chr2:105513439-105513539 |
| Pax6.P1. - 0.4kb | TAATGTCCGGCATTCAACAA | CTCTTGGGTCAGCTCAGTCC | chr2:105514389-105514520 |
| Pax6.P1. Promoter | ACTCCCGCCTCTTTTCTCTT | CCTAACTTCCCACCCCTTGT | chr2:105515078-105515189 |
| Pax6.P1. + 1.3kb | ACAGGAGCCTTGACAACGAC | CGAGAGGGGAGGAAAGAAGA | chr2:105516087-105516212 |
| Pax6.P1. + 1.7kb | GGCGATGCTTTTGTCCAG | AGTTCTGTGCCGGGAGAAT | chr2:105516526-105516649 |
| Pax6. P1. + 4.4kb | TCCCAGTTCTCAGGCAAGTT | CGGGTTTTGATTTGTTTTCC | chr2:105520034-105520133 |
| Pax6.Pα. Promoter | CGGAAAGGGTCGTTTTATCA | TGCATGGAAGGCTAATTGAA | chr2:105522398-105522525 |
| Pax6.Pα. +1.6.F | TCAGCTTGGTGGTGTCTTTG | CTGCAGAATTCGGGAAATG | chr2:105524001-105524115 |
| H3K4me3 Negative | GTGGCACATCACAAATGCTC | TCTCCAGTCTAACACTTGGCAAT | chr2:104646662-104646761 |
| Rcn1. + 5kb | TCCTGACACTTCTGCACTGG | TGGAACTTCAAGGTGTGCAG | chr2:105244709-105244812 |
| Rcn1. Exon 1 | GAGGACAACCAGAGCTTCCA | GCCTCTCCTTGCTCTCGTC | chr2:105239131-105239230 |

Supplementary Table 1: Sequences and positions of primers used in H3K4me3 ChIP.
